# NRF2 regulates the glutamine transporter Slc38a3 (SNAT3) in kidney in response to metabolic acidosis

**DOI:** 10.1038/s41598-018-24000-2

**Published:** 2018-04-04

**Authors:** Adam Lister, Soline Bourgeois, Pedro H. Imenez Silva, Isabel Rubio-Aliaga, Philippe Marbet, Joanne Walsh, Luke M. Shelton, Bettina Keller, Francois Verrey, Olivier Devuyst, Pieter Giesbertz, Hannelore Daniel, Christopher E. Goldring, Ian M. Copple, Carsten A. Wagner, Alex Odermatt

**Affiliations:** 1 Department of Pharmaceutical Sciences, Division of Molecular and Systems Toxicology, University of Basel, Klingelbergstrasse 50, 4056 Basel, Switzerland; 2 Institute of Physiology, Zürich Centre for Integrative Human Physiology, University of Zürich, Winterthurerstrasse 190, 8057 Zürich, Switzerland; 3 National Center for Competence in Research Kidney.CH, Zürich, Switzerland; 4 Department of Molecular and Clinical Pharmacology, MRC Centre for Drug Safety Science, University of Liverpool, Liverpool, L69 3GE UK; 5 Department of Biochemistry, ZIEL Research Center of Nutrition and Food Sciences, Technische Universität München, Freising, Germany

## Abstract

Expression of the glutamine transporter SNAT3 increases in kidney during metabolic acidosis, suggesting a role during ammoniagenesis. Microarray analysis of *Nrf2* knock-out (KO) mouse kidney identified *Snat3* as the most significantly down-regulated transcript compared to wild-type (WT). We hypothesized that in the absence of NRF2 the kidney would be unable to induce SNAT3 under conditions of metabolic acidosis and therefore reduce the availability of glutamine for ammoniagenesis. Metabolic acidosis was induced for 7 days in WT and *Nrf2* KO mice. *Nrf2* KO mice failed to induce *Snat3* mRNA and protein expression during metabolic acidosis. However, there were no differences in blood pH, bicarbonate, pCO_2_, chloride and calcium or urinary pH, ammonium and phosphate levels. Normal induction of ammoniagenic enzymes was observed whereas several amino acid transporters showed differential regulation. Moreover, *Nrf2* KO mice during acidosis showed increased expression of renal markers of oxidative stress and injury and NRF2 activity was increased during metabolic acidosis in WT kidney. We conclude that NRF2 is required to adapt the levels of SNAT3 in response to metabolic acidosis. In the absence of NRF2 and SNAT3, the kidney does not have any major acid handling defect; however, increased oxidative stress and renal injury may occur.

## Introduction

The kidney plays a major role in maintaining pH homeostasis and protecting against systemic pH disruption by excreting acids, reclaiming filtered bicarbonate (HCO_3_^−^) and by de novo synthesis of HCO_3_^−^ to compensate for HCO_3_^−^ used by metabolism^1^. HCO_3_^−^ synthesis in the proximal tubule is mainly generated by ammoniagenesis, in a process that also facilitates the excretion of protons from the proximal tubule in the form of ammonium (NH_4_^+^)^1^. In the collecting duct, excreted ammonia (NH_3_) acts as urinary buffer for secreted protons in the filtrate, which allows for further proton excretion by vacuolar H^+^-ATPases^1–4^. Ammoniagenesis is a multistep, enzymatically driven process utilizing the amino acid glutamine to yield two molecules of NH_3_ and two molecules of HCO_3_^−^ ^5^. Metabolic acidosis stimulates renal metabolism of glutamine, while concomitantly the level of glutamine in the blood decreases^5,6^. Specifically, glutamine is converted to glutamate, a process facilitated by phosphate-dependent glutaminase (GLS) to yield one molecule of NH_4_^+^. Glutamate is further converted to α-ketoglutarate by glutamate dehydrogenase (GDH) producing an additional NH_3_ molecule. The α-ketoglutarate is eventually directed into the tricarboxylic acid (TCA) cycle, yielding one molecule of HCO_3_^−^, and the oxaloacetate derivate is directed into the gluconeogenic pathway, a process facilitated by cytosolic phosphoenolpyruvate carboxykinase (PEPCK), which yields the final molecule of HCO_3_^−^ ^5^.

Under metabolic acidosis net extraction of glutamine from the plasma reaches 35%, a number that is beyond what is normally filtered by the kidney, suggesting that basolateral glutamine influx transporters contribute substantially to cellular glutamine supply^5^. Indeed, it has been suggested that during acute acidosis the direction in which the basolateral glutamine exchange transporter LAT2–4F2hc directs glutamine flux may be to favor intracellular import^5,7^. During chronic metabolic acidosis, the expression of the glutamine transporter *Snat3* (*Slc38a3*) is induced in parallel with other pH sensitive genes involved in glutamine metabolism, i.e. *Gls* and *Pepck*^8–13^. In *Snat3* knock-out (KO) mice, urinary NH_4_^+^ excretion was reduced, further supporting its potential role in renal ammoniagenesis^7,14^.

The transcription factor nuclear factor erythroid 2-related factor 2 (NRF2) regulates the basal and inducible expression of a battery of cell defense genes and serves as a mechanism for cells to resist chemical and oxidative stress^15,16^. Under baseline conditions, the Kelch-like ECH-associated protein 1 (KEAP1) targets NRF2 for ubiquitylation and subsequent proteasomal degradation^17^. In response to cellular stress, the interaction between NRF2 and KEAP1 is disrupted, resulting in the nuclear accumulation of de novo synthesized NRF2, where it binds and activates antioxidant response elements (AREs) in the promoter regions of target genes, such as NAD(P)H dehydrogenase quinone 1 (NQO1) and Glutamate-Cysteine Ligase Catalytic Subunit (GCLC)^16,18^. More recently, additional mechanisms for controlling the activity of NRF2 have been described, which include alternative interacting partners, transcriptional upregulation by a plethora of transcription factors which bind to specific sites within the *Nrf2* promoter, and post-translational modifications^19^. Apart from regulation of cell defense, it has become apparent that NRF2 can influence intermediary metabolism and mitochondrial function^19,20^. With respect to glutamine metabolism, when NRF2 is constitutively activated, glutamine is preferentially ushered into glutathione synthesis and the TCA cycle^21^.

Recently, microarray and RT-PCR analyses of kidneys from *Nrf2* KO mice identified *Snat3* as the most significantly down-regulated transcript compared to wild-type (WT) kidney^22^. Since SNAT3 is suggested to play a major role in the supply of glutamine during chronic metabolic acidosis, we investigated under conditions of metabolic acidosis and in the absence of NRF2 whether SNAT3 is induced in the kidney in order to maintain an adequate supply of glutamine for ammoniagenesis.

## Results

### NRF2 regulates SNAT3 in the kidney upon metabolic acidosis

**Figure 1:**
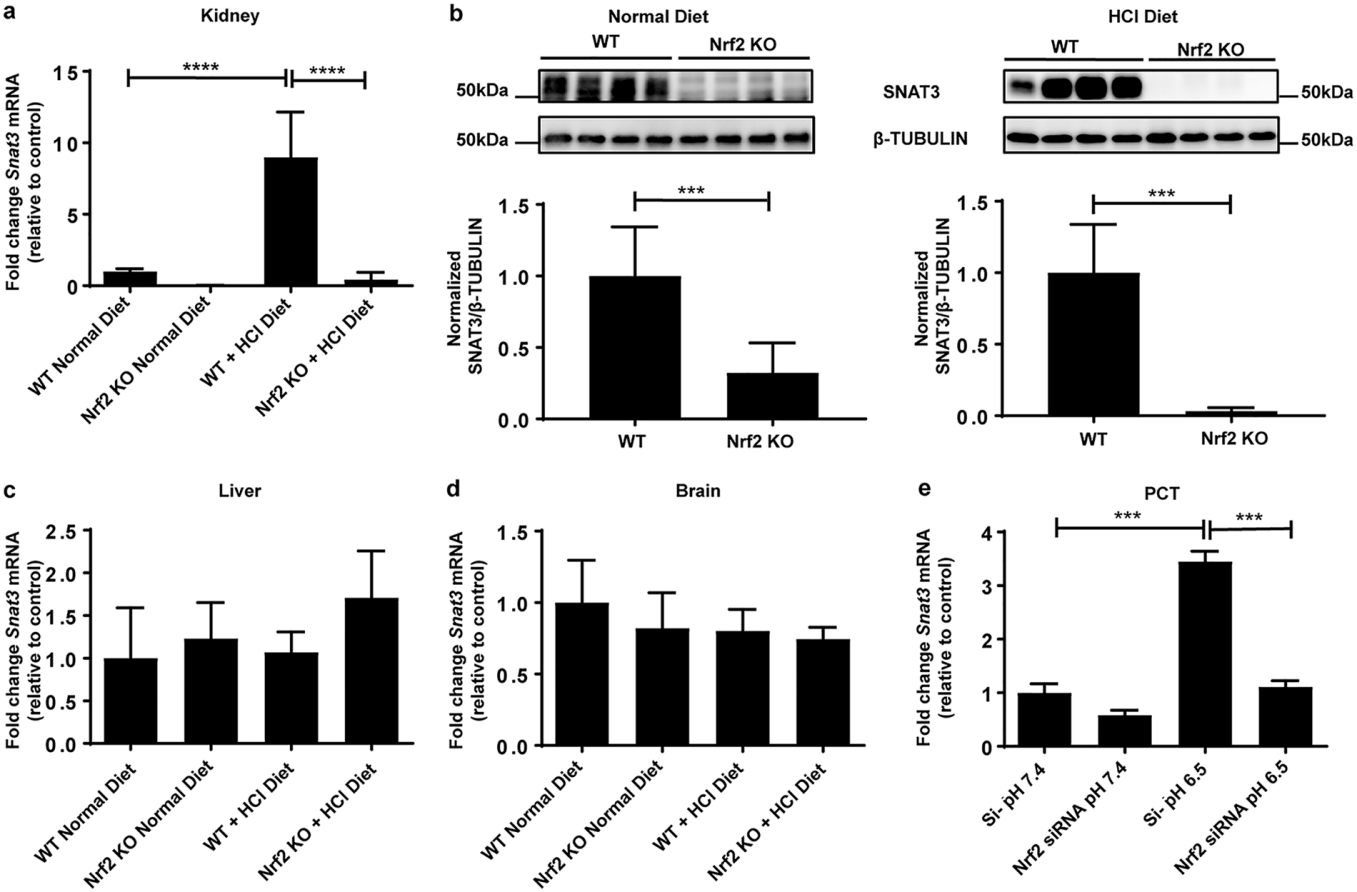
NRF2 regulates basal and metabolic acidosis induced SNAT3 levels in the kidney. WT and *Nrf2* KO mice were fed a normal diet or a HCl containing diet for 7 days. (**a**) qPCR analysis of *Snat3* mRNA expression in the kidney. *Snat3* mRNA levels were normalized to *Ppia*. (**b**) Immunoblot detection of SNAT3 using 20 µg total kidney membrane preparation. β-TUBULIN was used as a house-keeping control. (**c,d**) qPCR analysis of *Snat3* mRNA expression in liver and brain. (**e**) Primary proximal convoluted tubular cells (PCT) from WT kidneys were isolated, followed by depletion of *Nrf2* with targeted siRNA molecules and exposure of the cells to normal or acidic media (pH 7.4 or 6.5) for 24 h. qPCR analysis of *Snat3* mRNA expression in primary PCT. Data represent mean ± S.D. of n = 4–6 animals per group (**a**–**d**) or n = 4 independent PCT preparations (**e**). Statistical analysis for qPCR was performed with a one-way analysis of variance (with Tukey’s post test); ***P ≤ 0.001; ****P ≤ 0.0001. Statistical analysis for immunoblotting was performed with Student’s t-test; *a* = 0.05.

In agreement with our previous work^22^, we confirmed that kidneys from *Nrf2* KO mice express depleted levels of *Snat3* mRNA and significantly lower protein compared to WT kidneys (Fig. 1a,b and Supplemental Fig. S1). However, deletion of *Nrf2* did not affect Snat3 mRNA levels in liver and brain, suggesting that the *Nrf2*-dependent regulation was kidney-specific (Fig. 1c,d). Interestingly, *Nrf2* KO mice showed a trend to induce SNAT3 mRNA; however, they were unable to induce SNAT3 significantly in the kidney upon metabolic acidosis (Fig. 1a,b). Under baseline homeostatic conditions, SNAT3 is mostly expressed in the basolateral membrane of the S3 segment of the proximal tubule and extends to the S2 segment during metabolic acidosis^11–13^. To determine whether NRF2 is required for SNAT3 induction in the proximal tubule upon acid loading and if primary proximal convoluted tubular cells (PCT) could be utilized as a model to investigate the biological mechanisms of action of the SNAT3 transporter, we depleted *Nrf2* in PCT with targeted siRNA molecules and exposed the cells to acidic media at pH 6.5 or control media at pH 7.4. siRNA depletion of *Nrf2* caused a decrease in the mRNA and protein expression levels of *Nrf2* and of mRNA levels of the NRF2 target genes *Nqo1*, *Gstm1*, *Gsta3*, *Gclc* and *Gclm* at both pH 7.4 and pH 6.5 (Supplemental Fig. S2). PCT transfected with scrambled control siRNA showed a significant increase in *Snat3* mRNA expression following exposure to acidic media; whereas depletion of *Nrf2* abrogated the induction of Snat3 mRNA at pH 6.5 (Fig. 1e). However, unlike the *in vivo* study, we did not detect any SNAT3 protein expression under any of the conditions tested and therefore cannot utilize this model for further mechanistic testing (Supplemental Fig. S2). Also, although no morphological changes were observed upon incubating cells at lower pH, we cannot fully exclude the contribution of toxicity at the non-physiological pH of 6.5.

### *Nrf2* KO mice do not exhibit any major acid handling defects

Since SNAT3 has been considered a critical transporter in the proximal tubule to supply glutamine for ammoniagenesis, we next tested blood and urine acid-base parameters in WT and *Nrf2* KO mice under baseline conditions and after a dietary acid-loading. At baseline, *Nrf2* KO mice exhibited no major defect in acid handling compared to WT mice. However, plasma pH and bicarbonate showed a trend to be lower while plasma chloride tended to be elevated in *Nrf2* KO mice compared to WT (Table 1). Urinary pH was significantly more acidic in *Nrf2* KO mice with higher urinary ammonium as well as phosphate, titratable acidity and net acid excretion compared to the control group (Table 2, Supplementary Table S1). During the acute acid load, *Nrf2* KO mice were able to decrease their urinary pH and increased their urinary ammonium and phosphate excretion as much as the control mice (Table 2). Finally, after 7 days of dietary HCl load, blood pH, bicarbonate and pCO_2_ remained lower than under control diet in both groups of mice while blood chloride was increased (Table 1). Meanwhile, *Nrf2* KO mice could still acidify their urinary pH and significantly increased their urinary ammonium and net acid excretions as much as WT mice (Table 2). Phosphate as well as titratable acid excretions were lower than at baseline after 7 days HCl load in both genotypes; however, *Nrf2* KO mice excreted more phosphate and titratable acids than WT mice when normalized for creatinine. However, when normalized for 24 h urine volume, the difference for titratable acids and phosphate disappeared (Supplementary Table S1). Since urine volumes were small, sampling may not have been complete and quantitative in all animals. On the other hand, normalization to creatinine may also induce a bias as creatinine is actively secreted into urine and the role of Nrf2 in this process unknown. Nevertheless, the reduction in SNAT3 induction during metabolic acidosis in *Nrf2* KO mice was not associated with a failure to adapt to an acid-load.

**Table 1:** Blood values in *C57Bl*6 and *Nrf2 KO* mice under normal diet and during a 7 days acid load

	Baseline		7 days HCl	
	*WT* *N* = 6	*Nrf2 KO* *N* = 6	*WT* *N* = 6	*Nrf2 KO* *N* = *6*
pH	7.34 ± 0.01	7.29 ± 0.07	7.22 ± 0.08^##^	7.18 ± 0.12^##^
pCO_2_ (mmHg)	47.7 ± 2.8	46.1 ± 1.0	40.6 ± 3.3^##^	42.1 ± 3.5^##^
HCO_3_^−^ (mM)	24.9 ± 1.8	21.72 ± 3.36	15.92 ± 2.27^##^	15.28 ± 3.28^##^
pO_2_ (mmHg)	57.8 ± 6.7	61.3 ± 8.6	58.7 ± 1.9	62.9 ± 1.7
Na^+^ (mM)	155.2 ± 7.8	153.3 ± 2.0	153.7 ± 1.5	154.3 ± 2.4
Cl^−^ (mM)	116.2 ± 0.7	118.7 ± 2.6	123.3 ± 2.2^###^	123.5 ± 3.8^###^
Ca^2+^ (mM)	1.29 ± 0.03	1.32 ± 0.02	1.40 ± 0.05^#^	1.40 ± 0.07^#^

Summary of blood data obtained from WT and *Nrf2* KO mice fed a normal diet (control) or a HCl-containing standard diet for 7 days (acid). Data represent mean ± S.D. of n = 6 animals per group. Statistical analysis was performed with a one-way analysis of variance (with Tukey’s post test); *P ≤ 0.05; **P ≤ 0.01; ***P ≤ 0.001 for differences between genotypes for the same treatment and ^#^P ≤ 0.05; ^##^P ≤ 0.01; ^###^P ≤ 0.001 for differences between untreated and treated mice for the same genotype.

**Table 2:** Weight, food intake and urinary values in *C57Bl6* and *Nrf2 KO* mice under normal diet and a 2 or 7 days HCl load

	Baseline		2 days HCl		7 days HCl	
	*WT* *N* = 12	*Nrf2 KO* *N* = 12	*WT* *N* = 6	*Nrf2 KO* *N* = 6	*WT* *N* = 6	*Nrf2 KO* *N* = 6
Weight (g)	25.1 ± 1.9	23.0 ± 0.6*	26.0 ± 1.1	24.6 ± 0.7*	24.2 ± 0.9^#^	22.7 ± 1.1^#^
Food intake (g/24 h/body weight)	0.30 ± 0.06	0.40 ± 0.06 *	0.32 ± 0.05	0.34 ± 0.04	0.29 ± 0.01	0.30 ± 0.03^#^
Water intake (ml/24 h)	2.2 ± 1.1	1.5 ± 0.7*	2.5 ± 1.7	2.1 ± 0.9	4.8 ± 2.6^#^	5.5 ± 2.6^#^
Urine						
Volume (ml/24 h)	2.4 ± 0.5	1.8 ± 0.5*	2.3 ± 0.7	2.5 ± 0.3^#^	2.1 ± 0.8	2.8 ± 0.9^#^
Creatinine excretion (µmol/24 h)	6.4 ± 0.7	5.7 ± 1.2	6.3 ± 1.2	6.8 ± 0.9	4.6 ± 1.9	6.5 ± 1.0
Urinary pH	6.3 ± 0.2	5.9 ± 0.3*	5.6 ± 0.1	5.4 ± 0.1	5.6 ± 0.5	5.3 ± 0.1
NH_4_/Crea (mEq/mmol)	11.8 ± 12.3(6)	22.9 ± 16.2*(6)	71.5 ± 7.4^#^	78.7 ± 13.8^#^	140.2 ± 14.5^#^	133.7 ± 23.9^#^
TA/Crea (mEq/mmol)	14.9 ± 2.8(6)	22.4 ± 4.7*(6)	ND	ND	9.0 ± 5.1^#^	14.0 ± 0.8*^#^
NAE/Crea (mEq/mmol)	26.5 ± 9.9(6)	50.1 ± 14.8*(6)	ND	ND	149.1 ± 10.7	147.7 ± 23.7
Pi/Crea (mEq/mmol)	7.5 ± 0.6	8.8 ± 1.0*	22.7 ± 4.2^#^	24.2 ± 3.6^#^	2.3 ± 0.6^#^	3.4 ± 0.5*^#^
Ca^2+^/Crea (mEq/mmol)	0.19 ± 0.4	0.29 ± 0.08*	ND	ND	3.4 ± 1.0^#^	3.8 ± 1.2^#^
Na^+^/Crea (mEq/mmol)	25.9 ± 2.7	27.7 ± 6.3	ND	ND	35.5 ± 3.5^#^	41.0 ± 3.8*^#^
Cl^−^/Crea (mEq/mmol)	48.8 ± 7.0	50.4 ± 10.0	ND	ND	213.7 ± 15.7^#^	209.2 ± 25.4^#^
K^+^/Crea (mEq/mmol)	69.7 ± 9.4	70.2 ± 10.0	ND	ND	78.6 ± 6.2	77.1 ± 7.2

Summary of urinary data obtained from WT and *Nrf2* KO mice fed a normal diet (control) or a HCl-containing standard diet for 2 and 7 days (acid). Data represent mean ± S.D. of n = 6 animals per group. Statistical analysis was performed with a one-way analysis of variance (with Tukey’s post test); *P ≤ 0.05; **P ≤ 0.01; ***P ≤ 0.001 for differences between genotypes for the same treatment and ^#^P ≤ 0.05; ^##^P ≤ 0.01; ^###^P ≤ 0.001 for differences between untreated and treated mice for the same genotype.

### NRF2 does not affect the expression of ammoniagenic and gluconeogenic enzymes induced by metabolic acidosis

**Figure 2:**
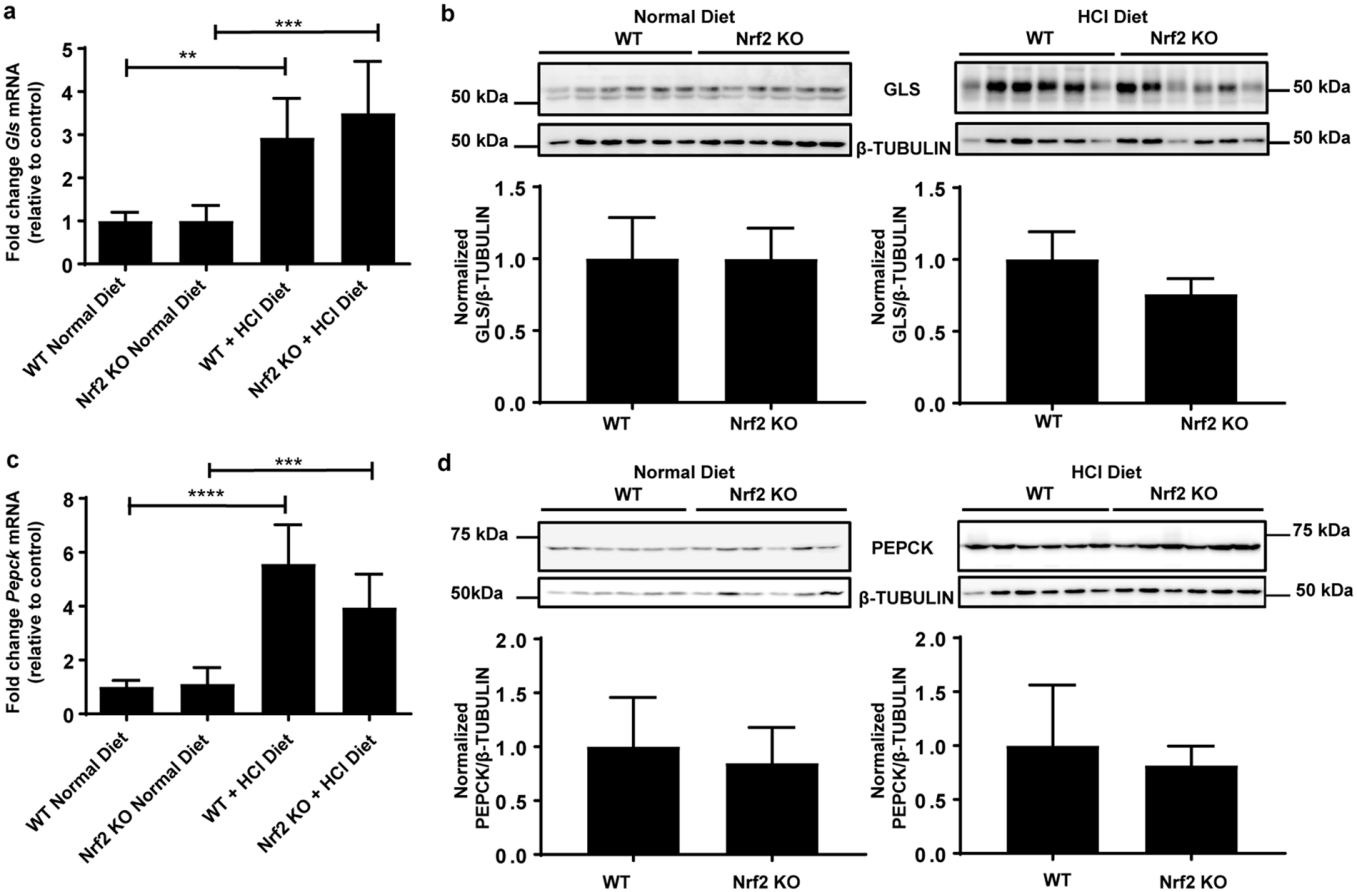
Ammoniagenic and gluconeogenic enzymes induced by metabolic acidosis are not changed by *Nrf2* deficiency. WT and *Nrf2* KO mice were fed a normal diet or a HCl-containing standard diet for 7 days. (**a**) qPCR analysis of *Gls* in the kidney. (**b**) Immunoblot detection of GLS using 40 µg total kidney homogenate. (**c**). qPCR analysis of *Pepck* mRNA expression in the kidney (**d**). Immunoblot detection of PEPCK using 40 µg total kidney homogenate. mRNA values were normalized to *Ppia* and β-TUBULIN was used as a house-keeping control in immunoblots. Data represent mean ± S.D. of n = 6 animals per group. Statistical analysis was performed with a one-way analysis of variance (with Tukey’s post test) for mRNA data and Student’s t-test comparing Nrf2 KO and WT for protein data; **P ≤ 0.01; ***P ≤ 0.001; ****P ≤ 0.0001.

In addition to SNAT3, the mRNA of enzymes involved in ammoniagenesis and subsequent gluconeogenesis, GLS and PEPCK, respectively, were also up-regulated in response to metabolic acidosis (Fig. 2a–d). However, no differences were observed in mRNA and protein expression of *Gls* and *Pepck* in *Nrf2* KO mice upon metabolic acidosis compared to WT control (Fig. 2a–d).

### *Nrf2* KO mice exhibit altered amino acid profiles and amino acid transporter expression upon metabolic acidosis

Although we found that NRF2 was indispensable for SNAT3 induction upon metabolic acidosis both *in vivo* and *in vitro*, we did not observe any major defect in ammonium excretion in the kidney. Since SNAT3 has been postulated to be the major upregulated basolateral glutamine transporter in the kidney during chronic metabolic acidosis, we explored possible mechanisms underlying the ability of the kidney to compensate for its loss in *Nrf2* KO mice. We first checked for differences in the amino acid profile in plasma and kidney of *Nrf2* KO compared to WT upon metabolic acidosis. The profile in plasma and kidney of *Nrf2* KO mice differed for several amino acids from WT mice (Supplementary Tables S2 and S3). From the 30 amino acids and derivatives measured in plasma glutamine, glutamate, histidine, phenylalanine, 3-methylhistidine and GABA were significantly increased in the *Nrf2* KO mice when compared to WT mice. Glutamine and histidine are substrates of SNAT3, and glutamate and GABA are involved in glutamine metabolism, which could explain the altered profile observed in the plasma of *Nrf2* KO mice. In kidney, from the 33 amino acids and derivatives measured histidine, glycine and beta-alanine were decreased and phenylalanine, valine, anserine and carnosine were significantly increased in the *Nrf2* KO mice when compared to WT mice. No changes between genotypes could be observed in the glutamine, glutamate and GABA concentrations.

**Figure 3:**
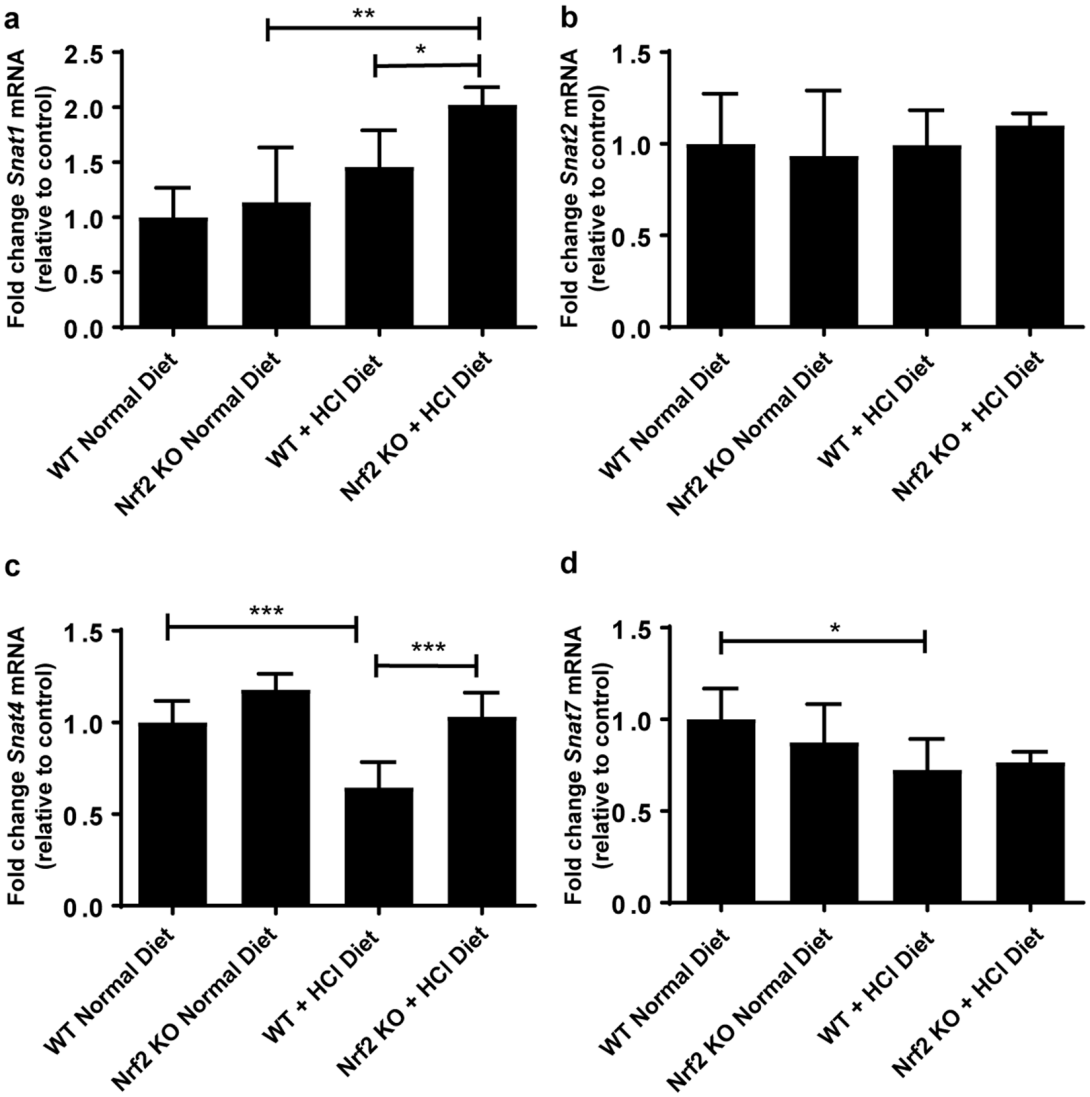
Snat/Slc38 transporter family members in the kidney may compensate for the loss of *Snat3* in *Nrf2* KO kidney. WT and *Nrf2* KO mice were fed a normal diet or a HCl-containing standard diet for 7 days. (**a**–**d**) qPCR analysis of *Snat1, 2, 4* and *7* in kidney. mRNA values were normalized to *Ppia*. Data represent mean ± S.D. of n = 6 animals per group. Statistical analysis was performed with a one-way analysis of variance (with Tukey’s post test); *P ≤ 0.05; **P ≤ 0.01; ***P ≤ 0.001.

**Figure 4:**
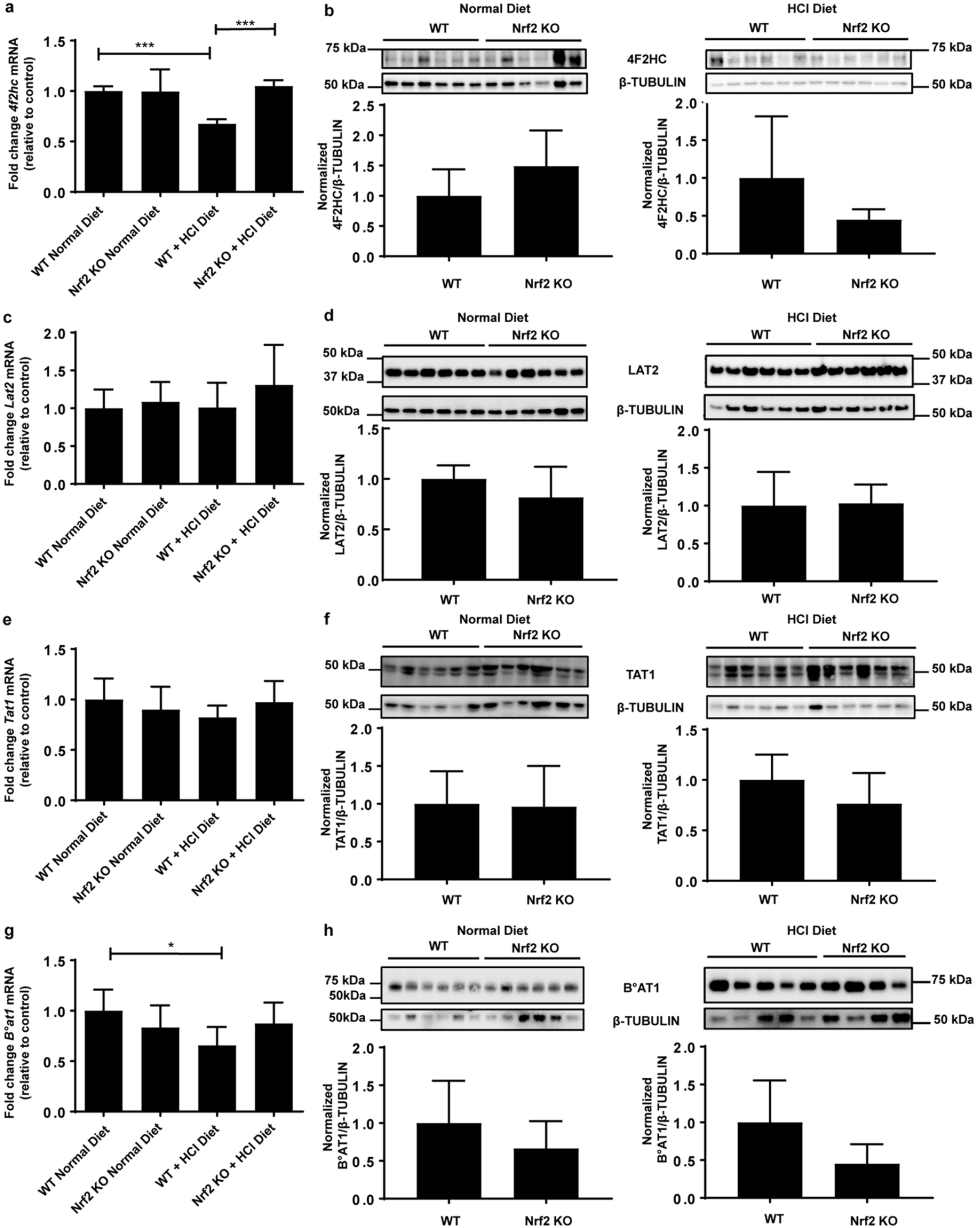
Amino acid transporter levels in the kidney remain unchanged in *Nrf2* KO kidney upon metabolic acidosis. WT and *Nrf2* KO mice were fed a normal diet or a HCl-containing standard diet for 7 days. qPCR and immunoblotting of (**a**,**b**) *4f2hc*, (**c**,**d**) *Lat2*, (**e**,**f**) *Tat1*, and (**g**,**h**) *B°at1* in the kidney. Immunoblot detection for 4F2hc, LAT2 and TAT1 were performed with 20 µg total kidney membrane preparation and for B°AT1 with 15 µg brush border membrane preparation. mRNA values were normalized to *Ppia*. β-TUBULIN was used as a house-keeping control for the immunoblots. Data represent mean ± S.D. of n = 6 animals per group. Statistical analysis was performed with a one-way analysis of variance (with Tukey’s post test); *P ≤ 0.05; ***P ≤ 0.001. Statistical analysis for immunoblotting was performed with Student’s t-test; *a* = 0.05.

Next, we examined a panel of genes encoding transporters which handle glutamine and other amino acids that can fuel ammoniagenesis (*Snat 1,2,4*, and 7 (*Slc38a1,2,4, and 7*), *b*^*0*,+^*at* (*Slc7a9*), *y*^+^*Lat1* (*Slc7a7*), *4F2hc* (*Slc3a2*), *Tat1* (*Slc16a1*0), *Lat2* (*Slc7a8*), and *B*^*0*^*at1* (*Slc6a19*)), transport ammonium (*Rhcg*, *Nkcc2, Nhe*4), protons (*Nhe3)* (*Slc9a3* and *4*), and calcium (*Ncx1*, *Trpv5*, *Calb1* and *Pmca4*). *Snat1* mRNA was significantly upregulated in *Nrf2* KO kidney upon metabolic acidosis (Fig. 3a). None of the other members of the Snat (Slc38) family tested were upregulated at mRNA level by metabolic acidosis (Fig. 3b–d). The mRNA expression of the amino acid transporters *b*^*0*,+^*at* and *y*^+^*lat1* were significantly suppressed upon metabolic acidosis in WT kidney, in agreement with our previous work^12^, whereas the expression remained unchanged in *Nrf2* KO kidneys (Supplementary Fig. S3). The same pattern was observed for 4F2hc (Fig. 4a). No change in mRNA expression was observed for *Lat2, Tat1*, and *B*^0^*at1*. (Fig. 4c,e,g). At protein level, no difference was observed between WT and *Nrf2* KO at baseline and in acid loaded conditions for 4F2hc, LAT2, TAT1 and B^0^AT1 (Fig. 4b,d,f,h). Additionally, no change in mRNA levels was observed for the ammonia transporter (*Rhcg*), the ion and ammonium transporter *Nkcc2*, and the Na^+^/H^+^ exchanger *Nhe3* (Supplementary Fig. S3). However, the mRNA levels of the Na^+^/H^+^ exchanger *Nhe4* were significantly up-regulated in kidneys from *Nrf2* KO mice under metabolic acidosis (Supplementary Fig. S3). Furthermore, no change was observed in the mRNA expression of proteins involved in active renal calcium handling, namely *Ncx1*, *Trpv5*, *Calb1*, and *Pmca4A* (Supplementary Fig. S4).

### Metabolic acidosis induces NRF2 activity in WT kidney

**Figure 5:**
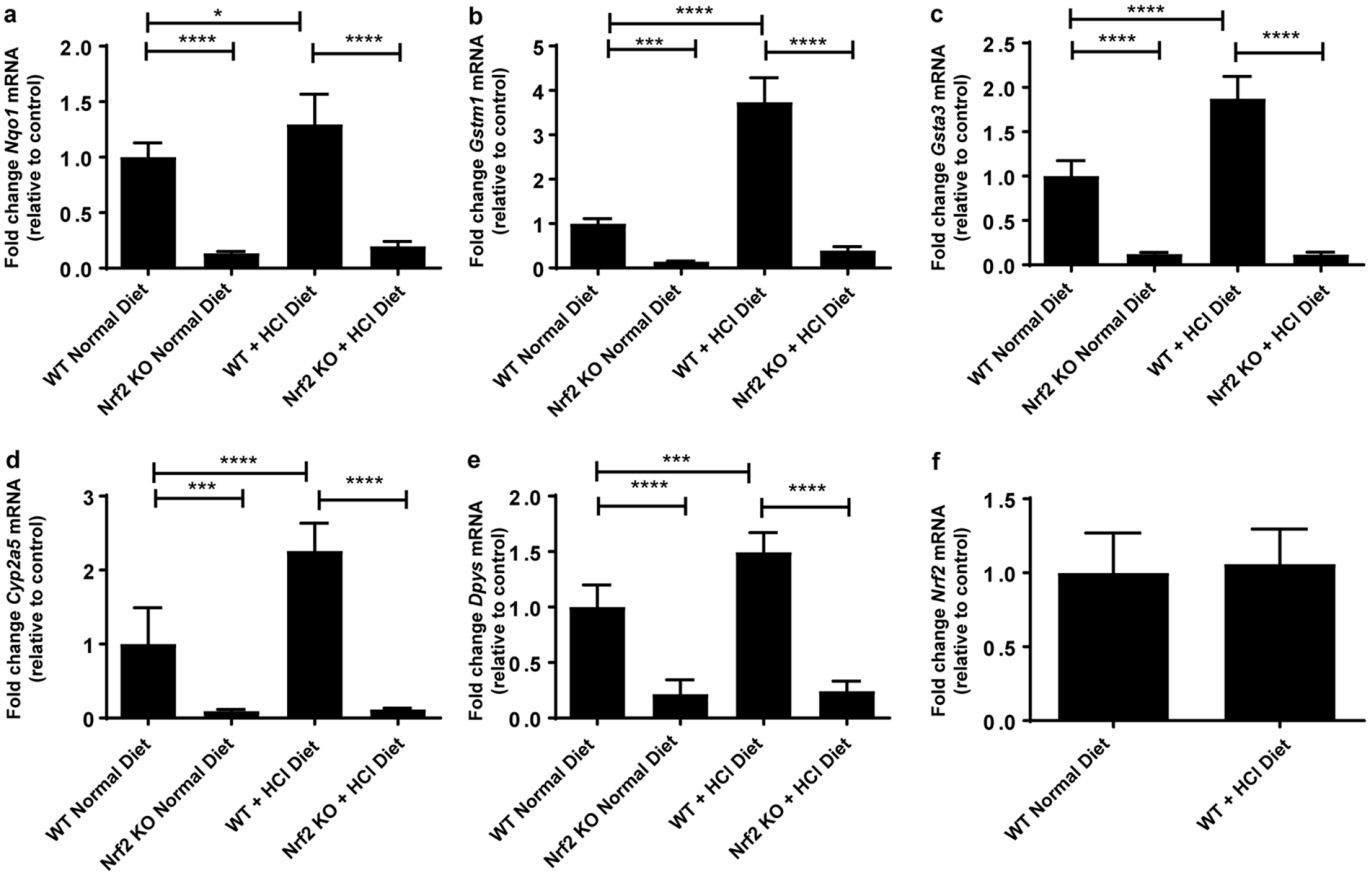
NRF2 activity is induced upon metabolic acidosis. WT and *Nrf2* KO mice were fed a normal diet or a HCl-containing standard diet for 7 days. (**a**–**f**) qPCR analysis of Nrf2 target genes *Nqo1*, *Gstm1*, *Gsta3*, *Cyp2a5*, *Dyps* and *Nrf2* in the kidney. mRNA values were normalized to *Ppia*. Data represent mean ± S.D. of n = 6 animals per group. Statistical analysis was performed with a one-way analysis of variance (with Tukey’s post-test); *P ≤ 0.05; ***P ≤ 0.001; ****P ≤ 0.0001.

**Figure 6:**
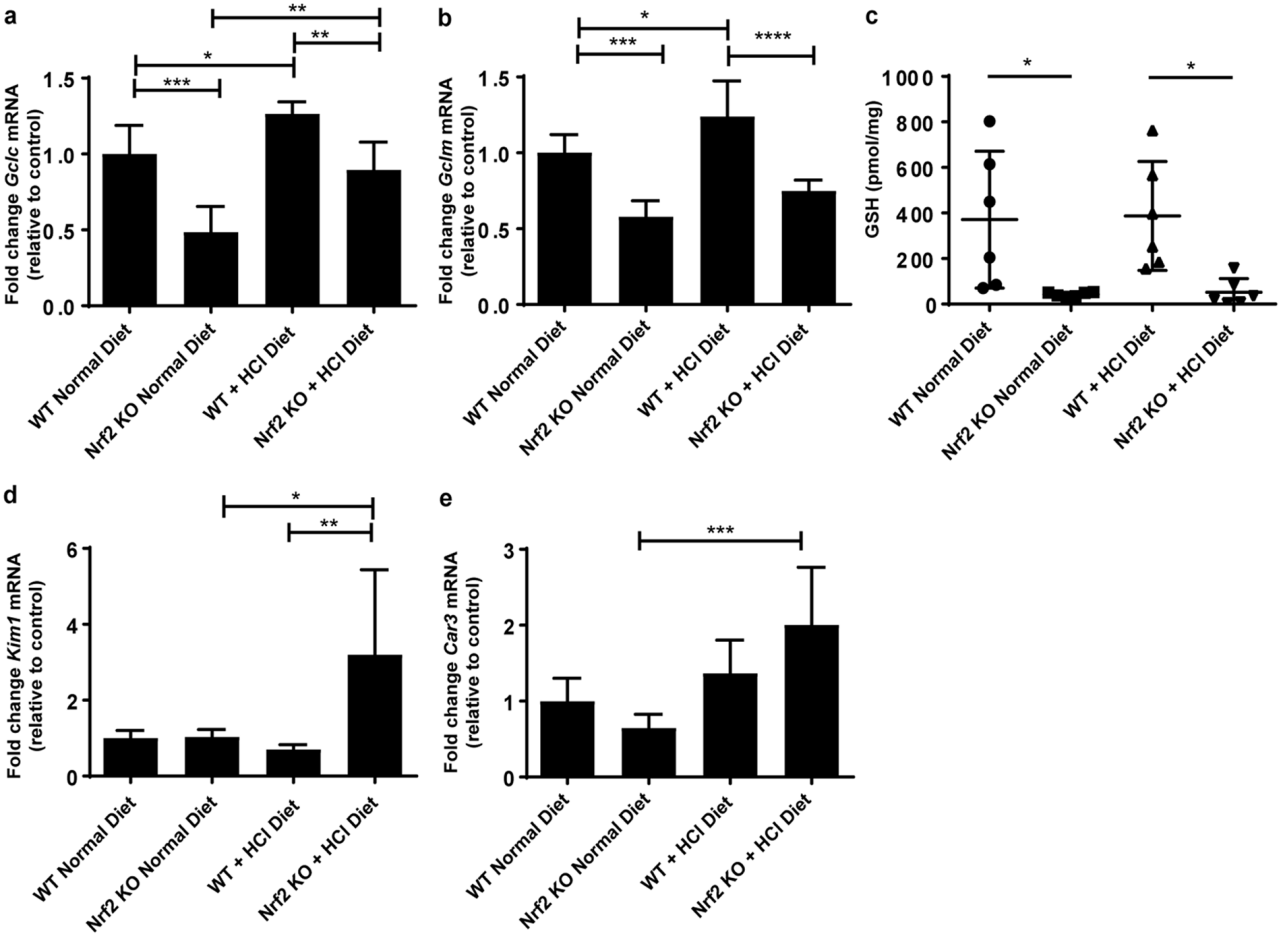
Metabolic acidosis increases oxidative stress in *Nrf2* KO kidney. WT and *Nrf2* KO mice were fed a normal diet or a HCl-containing standard diet for 7 days. qPCR analysis of the rate limiting glutathione synthesis enzyme, GSL, subunits (**a**) *Gclc* and (**b**) *Gclm*. (**c**) total glutathione contents of kidney tissue, normalized to tissue weight. qPCR analysis of kidney proximal tubule oxidative stress markers (**d**), *Kim1* and (**e**), *Car3*. mRNA values were normalized to *Ppia*. Data represent mean ± S.D. of n = 6 animals per group. Statistical analysis was performed with a one-way analysis of variance (with Tukey’s post test); *P ≤ 0.05; **P ≤ 0.01; ***P ≤ 0.001; ****P ≤ 0.0001.

In order to determine whether metabolic acidosis could induce NRF2 activity we measured a panel of its target transcripts. Accordingly, the mRNA levels of *Nqo1*, Glutathione S-Transferase Mu 1 (*Gstm1*), Glutathione S-Transferase Alpha 3 (*Gsta3*), Cytochrome P450 Family 2 Subfamily A Member 5 (*Cyp2a5*), Dihydropyrimidinase (*Dpys*), Glutamate-Cysteine Ligase Modifier Subunit (*Gclm*), and *Gclc* were significantly upregulated upon metabolic acidosis and glutathione (GSH) levels were maintained at levels similar to those in WT animals on a normal diet (Figs 5a–e and 6a–c). *Nrf2* mRNA levels remained unchanged suggesting that regulation of NRF2 activity by metabolic acidosis occurs on a posttranscriptional level (Fig. 5f). In agreement with our previous study, we found a significant down regulation of the Nrf2 target genes *Nqo1*, *Gstm1*, *Gsta3*, *Cyp2a5, Dpys*, *Gclm*, and *Gclc* in *Nrf2* KO kidneys (Figs 5a–e and 6a,b) and a subsequent reduction in the stimulation of their mRNA expression during metabolic acidosis^22^. Also, GSH levels were low in *Nrf2* KO kidneys at baseline and remained almost unmeasurable during metabolic acidosis (Fig. 6c). The absence of induction of transcripts involved in the defense against oxidative stress during metabolic acidosis and the very low levels of GSH in *Nrf2* KO kidneys were associated with a significant increase in the levels of the proximal tubule specific kidney injury and oxidative stress markers *Kim1* (*Havcr1*) and carbonic anhydrase III(*Car3*), respectively (Fig. 6d,e)^23,24^.

## Discussion

In this study, we have shown for the first time that Nrf2 regulates the induction of the renal glutamine transporter SNAT3 (*Slc38a3*) upon metabolic acidosis. Further, we confirmed our previous observation that NRF2 also controls basal SNAT3 expression in mouse kidney^22^. Importantly, we found that NRF2 does not control basal *Snat3* mRNA expression in the liver and brain, and identified that both organs are insensitive to *Snat3* induction upon metabolic acidosis, suggesting a kidney-specific mechanism of control.

With respect to the current knowledge of the transcriptional regulation of *Snat3*, work by Balkrishna *et al*. demonstrated that tissue-specific S*nat3* expression is predominantly controlled by various epigenetic factors^25^. Importantly, they also identified two SP1 binding sites, one important for basal *Snat3* promoter transactivation and a second pH sensitive SP1 binding site to upregulate *Snat3* under metabolic acidosis^25^. Our data now indicate that NRF2 is also critical for the transcriptional control of *Snat3* in mouse kidney. Evidence is increasing that NRF2 forms complexes with the transcription factor SP1, which directly affect the regulation of SP1 and NRF2 target genes^26,27^. Furthermore, it has been reported that NRF2 and the SP family proteins, including SP1, synergistically enhance the expression of the ion transporters, colonic H/K-ATPase and kNBC1, as shown in transfected HEK-293T and CV-1 kidney cells under potassium-depleted conditions^28^. This effect was abolished upon transfection with dominant negative NRF2^28^. Importantly, chromatin immunoprecipitation showed that NRF2 and SP1 bound to the promoter of kNBC1, yet only SP1 bound to the promoter of colonic H/K-ATPase, suggesting the formation of a NRF2-SP1 complex in certain situations^28^. The hypothesis that *Snat3* transcription may be regulated by a NRF2-SP1 complex warrants further investigation.

The SLC38 family of amino acid transporters, to which SNAT3 (SLC38A3) belongs, is split into two major subgroups: system A and system N^14^. SNAT3 belongs to the system N transporters together with SNAT5 and the more recently characterized SNAT7^29^. Each transporter has specific activities for the amino acids glutamine, histidine and asparagine. During metabolic acidosis, both glutamine and histidine levels in blood were increased in *Nrf2* KO mice as compared to WT mice, whereas in kidney tissue of *Nrf2* KO mice glutamine remained the same and histidine levels were significantly reduced. The changes in plasma might be a direct effect of reduced SNAT3 expression but suggest that glutamine levels in the kidney are maintained at normal levels by other mechanisms in the absence of SNAT3. We observed no difference in the mRNA levels of the N-type transporters *Snat5 and Snat*7 in the kidneys of Nrf2 KO mice, therefore it is unlikely that these transporters are involved in a compensatory mechanism. The characterized system A transporters; SNAT1, SNAT2 and SNAT4 can carry glutamine but prefer small neutral amino acids^14^. Interestingly, our data show that *Snat1* mRNA is significantly upregulated in *Nrf2* KO mice upon metabolic acidosis, whilst in the whole animal *Snat3* KO mouse, SNAT1 protein was profoundly elevated in the brain^7^. Although the fold induction of *Snat1* was modest, it may contribute to elevating kidney glutamine levels during metabolic acidosis when SNAT3 is lost. The exact localization of SNAT1 in kidney, however, is currently unknown. We also observed a significant induction of the sodium/proton exchanger *Nhe4* (*Slc9a4*) in the *Nrf2* KO mouse kidney following metabolic acidosis. NHE4 is critical for ammonia transport in the thick ascending limb of the loop of Henle, interstitial ammonia accumulation, and maintenance of systemic pH^30^. Its upregulation in *Nrf2* KO mice may serve compensatory functions.

Metabolic acidosis results in the upregulation of enzymes which direct the metabolism of glutamine towards ammoniagensis (GLS and glutamate dehydrogenase 1) and gluconeogenesis (PEPCK). When working efficiently, both pathways combine to produce two NH_3_ and two HCO_3_^−^ ions per glutamine molecule^5^. NRF2 has also been shown to influence the direction of the metabolism of glutamine in favor of GSH biosynthesis and the TCA cycle^21^. In addition, NRF2 can inhibit gluconeogenesis, although the mechanism remains to be fully understood^19^. We show that GSH levels are significantly reduced in the *Nrf2* KO kidney compared to WT, probably due to the downregulation of GCLC and GCLM, which code for the two subunits of the rate limiting enzyme in glutathione synthesis, GCL^21^. We show that the mRNA levels of *Gclc* and *Gclm* are significantly induced and that GSH levels are maintained upon metabolic acidosis in WT kidney, which suggests that glutamine is being utilized for all the pathways mentioned above during metabolic acidosis. This would imply that an active NRF2 system regulates pathways that compete for glutamine in the kidney and therefore reduces the efficiency by which the proximal tubular cells can utilize glutamine for ammoniagenesis and gluconeogenesis. Therefore, it is possible that in the absence of NRF2 in this setting, the demand for glutamine is reduced since it is no longer fluxed into GSH biosynthesis. If this is the case, in the absence of SNAT3, intracellular demands for glutamine may be met by the induction of the SNAT1 transporter or additional mechanisms yet to be identified. This hypothesis has to be tested in future experiments.

The primary role of NRF2 is to coordinate the upregulation of antioxidant and detoxification genes to defend against the detrimental effects of oxidative stress^31^. The importance of this pathway, with respect to the kidney have been demonstrated to protect against a number of nephrotoxic insults in *Nrf2* KO animals^32^. Importantly, animal models of chronic kidney disease (CKD) showed reduced levels of NRF2 and its target genes^33–36^. Although this current investigation focused on SNAT3 and metabolic acidosis, it also gave us the opportunity to examine what happens to the NRF2 system in the kidney during metabolic acidosis. We show for the first time *in vivo* that the NRF2 target genes *Gclm*, *Gclc*, *Nqo1*, *Gstm1*, *Gsta3*, *Cyp3a5* and *Dyps* are significantly induced upon metabolic acidosis and that Nrf2 is needed to maintain GSH levels in the WT kidney under such conditions^22^. Thus, our data suggest that metabolic acidosis in this setting is an oxidative insult and that the kidneys baseline defense needs to be stimulated in a NRF2-dependent manner in order to deal with the perturbation in homeostasis. Indeed there is *in vitro* evidence that NRF2 is activated in the prostate cancer cell AT-1 following exposure to acidic media; however, the opposite was observed in the MCF-7 breast cancer cell line^37,38^. Additionally, in the *Nrf2* KO kidney mRNA levels of *Gclm*, *Gclc*, *Nqo1*, *Gstm1*, *Gsta3*, *Cyp2a5* and *Dyps* were significantly depleted, as well as total GSH, in agreement with our previous study^22^. Importantly, upon metabolic acidosis we observed a significant increase in the proximal tubular injury and oxidative stress markers *Kim1*^24^ and carbonic anhydrase type 3 (*Car3*)^23^ specifically in the *Nrf2* KO kidneys. Although the kidney does not have any major acid handling defects in this experimental setting after 7 days, this is an indicator that the loss of *Nrf2* may be detrimental to the function of the proximal tubule following long term exposure and should be investigated further. The data may also indicate that the role of SNAT3 in ammoniagenesis is less critical than suggested by previous data and that SNAT3 may rather contribute to the anti-oxidative defense by supplying substrates for GSH synthesis. Clearly, this will require further detailed analyses developing cell-specific *Snat3* deficient mouse models.

In summary, NRF2 regulates the levels of SNAT3 in response to metabolic acidosis. However, in the absence of SNAT3 induction, the kidney does not show any impairment in the ability to adapt acid-excretion to an acid-load. Compensatory adaption of other transporters in the kidney or metabolic redirection of glutamine away from glutathione synthesis may account for the loss of SNAT3 induction but this is associated with increased vulnerability to oxidative stress.

## Methods and Materials

### Animals

Male *Nrf2* KO mice, age 10–14 weeks old (C57BL/6 background, generation, and genotyping have been described previously^39,40^), were bred at the University of Liverpool Biomedical Services Unit and transferred to the Institute of Physiology, University of Zürich. Age and sex matched WT C57BL/6 mice were supplied by Janvier, France. All animal experiments were performed according to the Swiss Law of Animal Welfare and approved by the local authority (Veterinäramt Zurich).

For experiments, mice were housed in metabolic cages (Techniplast, Buguggiate, Italy). Mice were given water ad libitum and were fed with a standard powdered laboratory chow mixed with water (50/75 w/v) (Kliba, Augst, Switzerland) to adapt to metabolic cages for 2 days. Two sets of experiments were performed. The first set of 6 WT and 6 *Nrf2* KO mice received a standard diet for 2 days, during which 24 h urine samples were collected under light mineral oil in the urine collector to determine daily urinary parameters. After 2 days, before euthanasia, retro-orbital blood samples were taken under light anesthesia with isoflurane. Plasma, kidneys, brains and livers were harvested after euthanasia. The second set of 6 WT and 6 *Nrf2* KO mice received a standard diet during the first 24 h urine collection and were then switched to a dietary acid load for 7 days where mice were given a HCl-containing diet with 150 ml of 0.33 M HCl added to 100 g powdered standard food. Food, water intake, and urine excretion were monitored at day 0, 2 and 7 of the HCl diet. On the last day of experiments, before euthanasia, retro-orbital blood samples were taken under light anesthesia with isoflurane. Plasma, kidneys, brains and livers were harvested after euthanasia.

### Analytic procedures

Blood pH, pCO_2_, and electrolytes were measured with a pH/blood-gas analyzer (ABL77 Radiometer). Urinary pH was measured with a pH-meter (Metrohm AG, Canada) and creatinine by a modified kinetic Jaffé colorimetric method^41^. Urinary NH_4_^+^ was measured with the Berthelot protocol^42^. Urinary inorganic phosphate (Pi) concentration was determined by the phosphomolybdate method^43^. Urinary titratable acidity was measured according to Jorgensen and Siggaard-Andersen^44,45^. Briefly, CO_2_ was eliminated by hydrochloric acid addition. Then, titratable acidity was measured by sodium hydroxide (1 N) titration to pH 7.40 using DL 55 Mettler Toledo® titrator, ST20 Mettler® sample changer and Inlab® Semi micro pH electrode. Urinary pH was measured on fresh urine with a pH-meter. Citric acid was measured using a kit from Boehringer Mannheim/ R-Biopharm.

### Amino acid measurements

Quantitative amino acid measurements were performed using targeted LC-MS/MS based on the method described by Harder *et al*.^46^. Briefly, plasma samples (10 µl) were dissolved in 500 µl ice-cold methanol containing an internal standard mixture of 15 deuterated amino acids. After centrifugation (10 min, 10 °C, x g), samples were dried. Kidney tissue samples were extracted in 30x methanol mass using TissueLyser. Amino acids in plasma and kidney samples were derivatized to their butyl esters as described by Gucciardi *et al*.^47^. Briefly, a mixture of 95% n-butanol and 5% acetylchloride (v/v) was added to the dried samples. Subsequently, the samples were incubated at 60 °C for 15 minutes at 600 rpm (Eppendorf Thermomixer Comfort; Eppendorf, Hamburg, Germany). The samples were dried and reconstituted in a 200 µl mixture of methanol/water/formic acid (70/30/0.1% v/v).

The analysis was performed on a triple quadrupole QTRAP 5500 LC-MS/MS system operating in positive ESI mode (AB Sciex, Framingham, MA) equipped with a 1200 series binary pump (Agilent, Santa Clara, CA) and coupled to an HTC pal autosampler (CTC Analytics, Zwingen, Switzerland). Chromatographic separation was achieved using a Zorbax Eclipse XDB-C18 column (length 150 mm, internal diameter 3.0 mm, particle size 3.5 µm; Agilent). Analytes were measured in scheduled multiple reaction monitoring (MRM). For absolute quantification, a 10-point calibration was performed, using a mixture containing all amino acids in the measurement (A9906 amino acid standards, Sigma-Aldrich, Taufkirchen, Germany). Data analysis was done using Analyst 1.5.1® software (AB Sciex).

### Analysis of mRNA expression

For isolation of total RNA kidney tissue (n = 6 per group), the Direct-Zol RNA MiniPrep kit (Zymo Research, Freiburg, Germany) including on-column DNAse treatment was applied. RNA concentration was assessed using a NanoDrop ND 1000 (Fisher Scientific, Reinach, Switzerland). cDNA was synthesized from 2 μg total RNA using Superscript III reverse transcriptase (Invitrogen, Carlsbad, CA, USA) following the manufacturers protocol. Relative mRNA quantification was performed by real-time quantitative RT-PCR using a rotor-gene 6000 (Corbett Research, Sydney, Australia). Briefly, the cDNA (10 ng) was mixed with gene-specific primers (Microsynth, Balgach, Switzerland) (200 nM) (supplementary Table S4) and KAPA SYBR FAST qPCR reagent (Kapasystems, Boston, MA, USA) (5 μl), in a final volume of 10 μl. Thermal cycler parameters were as follows: 15 min at 95 °C, followed by amplification of cDNA for 40 cycles with melting for 15 s at 94 °C, annealing for 30 s at 56 °C and extension for 30 s at 72 °C. For each sample, three technical replicates were analyzed. Expression was normalized to cyclophylin A (*Ppia*) control. Fold changes were quantified as 2^−(ΔCt sample-ΔCt control)^, as described previously^48^.

### Immunoblotting

Total kidney homogenates were prepared by mechanical dispersion (Polytron Kinematica PT10/36, Switzerland) in 200 μl ice-cold resuspension buffer (200 mM Mannitol, 80 mM HEPES, 41 mM KOH, pH 7.5) containing EDTA-free proteinase inhibitor mix (Roche, Mannheim, Germany). The total membrane fraction was isolated from total homogenates by first centrifuging it at 2000 rpm (5415 R Centrifuge, Eppendorf, Hamburg, Germany) for 20 min at 4 °C. Next the supernatant was further centrifuged at 41000 rpm (Sorvall RC M120 EX Centrifuge, RP45A-0030 rotor) at 4 °C for 1 hour and the pellet was resuspended in resuspension buffer^7^.

Brush border membrane vesicles were prepared as described previously^49^. Briefly, mouse kidneys were homogenized in 2 ml of homogenization solution (300 mM Mannitol, 5 mM EGTA, 12 mM Tris, pH 7.1 containing EDTA-free proteinase inhibitor mix) followed by addition of 2.8 ml ice-cold distilled water and 58 μl of 1 M MgCl_2_. The mixture was kept for 15 min on ice and centrifuged at 4600 rpm for 15 min at 4 °C (5415 R Centrifuge). The supernatant was centrifuged at 16000 rpm for 30 min at 4 °C (Sorvall RC 5 C Plus centrifuge, SS34 rotor) and the pellet was resuspended in 2 ml membrane buffer (300 mM Mannitol, 20 mM HEPES, 12 mM Tris, pH 7.4, containing EDTA-free proteinase inhibitor mix). The mixture was centrifuged again at 16000 rpm for 30 min at 4 °C and the pellet resuspended in 200 µl membrane buffer.

Whole PCT cell lysates were obtained by lysing cells in 100 µl of ice cold RIPA buffer (Sigma, St. Louis, MO) supplemented with 1x complete mini protease inhibitors (Roche, Mannheim, Germany).

The total protein concentration was measured with the Bio-Rad DC Protein Assay (Bio-Rad, USA) and 10–40 µg of protein preparations diluted in Laemmli buffer were loaded onto SDS-PAGE electrophoresis gels with 8–10% polyacrylamide, followed by transfer onto polyvinylidene difluoride membranes (Immobilon-FL, Millipore, USA). Immunoblotting was performed by first blocking with 5% non-fat dry milk in TBS-Tween followed by 30 min washing and overnight incubation with primary antibodies. After additional washing steps, anti-mouse, anti-rabbit or anti-goat IgG horseradish peroxidase-conjugated antibodies at 1:5000 or 1:10000 dilutions were used and the chemiluminescent signal was generated using the HRP substrate Immobilon Western (Millipore, Billerica, MA, USA). Images were acquired with the Imagequant LAS-4000 (Fujifilm). We used as primary antibodies: rabbit anti-SNAT3 (1:1000)^50^, goat anti-SNAT3 (1:1000, sc-33445, Santa Cruz Biotechnology, Santa Cruz, CA, USA), rabbit anti-PEPCK (1:5000, Cayman Chemical, Ann Arbor, MI, USA), rabbit anti-GLS/PDG (1:5000, kindly provided by N. Curthoys, Colorado State University, USA), rabbit anti-TAT1 (1:500)^51^, mouse anti-4F2hc (1:1000)(Santa Cruz Biotechnology, Santa Cruz, CA, USA).), rabbit anti-B^0^AT1 (1:1000)^52^, rabbit anti-b^0,+^AT1 (1:1:2000)^53^, rabbit anti-rBAT (1:2000)^54^, kindly provided by Manuel Palacín, Universitat de Barcelona, Spain), and rabbit LAT2 (1:2000)^55^. Measured densitometric values for all proteins were corrected by mouse anti-β-TUBULIN (1:25000, Sigma, USA) densitometric values for samples from the *in vivo* study and anti-β-ACTIN (1:10000, Santa Cruz Biotechnology) densitometric values for samples from PCT experiments. If necessary, membranes were subjected to mild acid stripping in 0.2 M glycine, 0.1% SDS, 1% Tween 20, pH 2.2 twice for 5–10 minutes and washed twice with PBS and TBST before blocking and reprobing.

### Isolation of proximal tubular cells

Primary proximal tubular cells (PCT) were isolated from male C57/BL6 mice (7 to 14 weeks) as described previously^56^. Briefly, renal cortices were sliced in dissection solution (HBSS with 15 mM HEPES, 10 mM D-glucose, 5 mM glycine, 1 mM L-alanine buffered to pH 7.4 and osmolality of 325 mosmol/kgH_2_O) into pieces of approximately 1 mm^2^ followed by collagenase type II digestion (Worthington Biochemical, Lakewood, NJ) for 30 min at 37 °C in presence of soybean trypsin inhibitor (Sigma, St. Louis, MO). Then they were transferred onto a sieve tower with two pore sizes (250 µm and 80 µm) (Retsch, Haan, Germany). The proximal tubule fragments on the lower sieve were collected by flushing the sieve in the reverse direction with dissection solution containing 1% BSA. Upon centrifugation the fragments of two kidneys were resuspended in culture medium (1:1 DMEM/F12 without phenol red and supplemented with 15 mM HEPES, 0.55 mM Na-pyruvate, 100 × non-essential amino acids 10 ml/L, and REGM SingleQuot Kit Supplements & Growth Factors (Lonza, Verviers, Belgium) and distributed onto one 24 well plate and left unstirred for 72 h at 37 °C and 95% air-5% CO_2_ in a standard humidified incubator. After 3 days the medium was replaced and on day 7 the cells reached confluence and were used for further experiments.

### RNA interference

Short interfering RNA (siRNA) targeting mouse *Nrf2* (D-040766-01) and scrambled non targeting control (D-001810-03) were purchased from the Dharmacon siGENOME library (Dharmacon via Fisher Scientific, Reinach, Switzerland). Confluent isolated proximal tubules were transfected with 20 nM siRNA using Lipofectamine RNAiMAX (Invitrogen, Carlsbad, CA, USA) according to the manufacturer’s protocol. After incubation for 24 h, cells were exposed to media of adjusted to pH 7.4 or 6.5 for a further 24 h.

### Quantification of Glutathione

Kidney tissue (15–20 mg) was homogenized and total glutathione levels were determined using the GSH-Glo assay kit (Promega, Southampton, UK) following the manufacturer’s instructions.

### Statistics

Data are expressed as mean ± S.D. of 6 animals per group or 4 independent PCT isolations. The significance of differences within the data were assessed by one-way analysis of variance (with Tukey’s post-test) or Student’s t-test. Amino acids profiles in kidney and plasma were analyzed using the Mann-Whitney test as we cannot assume the assumption of normality in these datasets. A two-sided *p* value of ≤0.05 was considered to be statistically significant. Figures were generated using GraphPad Prism 6.

### Data Availability

All data generated or analyzed during this study are included in this published article (and its Supplementary Information files).

## Electronic supplementary material

Supplementary Information
