# Supplementary material for: NRF2 regulates the glutamine transporter Slc38a3 (SNAT3) in kidney in response to metabolic acidosis
Source: Sci Rep. 2018 Apr 4;8:5629. doi: 10.1038/s41598-018-24000-2 (PMC5884861; doi:10.1038/s41598-018-24000-2)
Supplement: Supplementary file 1 — Supplementary Information [file 41598_2018_24000_MOESM1_ESM.pdf]

Supplemental Information

NRF2 regulates the glutamine transporter Slc38a3 (SNAT3) in kidney in response to metabolic acidosis

Adam Lister[1,3+], Soline Bourgeois[2,3+], Pedro H. Imenez Silva[2,3+], Isabel Rubio-Aliaga[2,3], Philippe Marbet[1,3], Joanne Walsh[4], Luke M. Shelton[4], Bettina Keller[2], Francois Verrey[2,3], Olivier Devuyst[2], Pieter Giesbertz[5], Hannelore Daniel[5], Christopher E. Goldring[4], Ian M. Copple[4], Carsten A. Wagner[2,3*], Alex Odermatt[1,3*]

[1] Division of Molecular and Systems Toxicology, Department of Pharmaceutical Sciences, University of Basel, Klingelbergstrasse 50, 4056, Basel, Switzerland; [2] Institute of Physiology, Zürich Centre for Integrative Human Physiology, University of Zürich. Winterthurerstrasse 190, 8057 Zürich, Switzerland; [3]National Center for Competence in Research Kidney.CH, Switzerland, [4] MRC Centre for Drug Safety Science, Department of Molecular and Clinical Pharmacology, University of Liverpool, L69 3GE, UK [5]ZIEL Research Center of Nutrition and Food Sciences, Department of Biochemistry, Technische Universität München, Freising, Germany

+ contributed equally and share first authorship

* to whom correspondence should be addressed

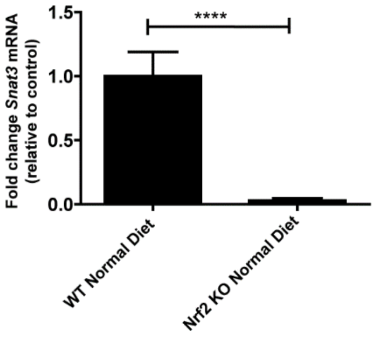

Supplemental Figure S1 Confirmation of *Snat3 depletion in Nrf2* KO kidney. WT and *Nrf2* KO mice were fed a normal diet and *Snat3* mRNA expression in the kidney was determined by qPCR analysis. Statistical analysis for qPCR was performed with a student's paired t-test ****P≤0.0001.

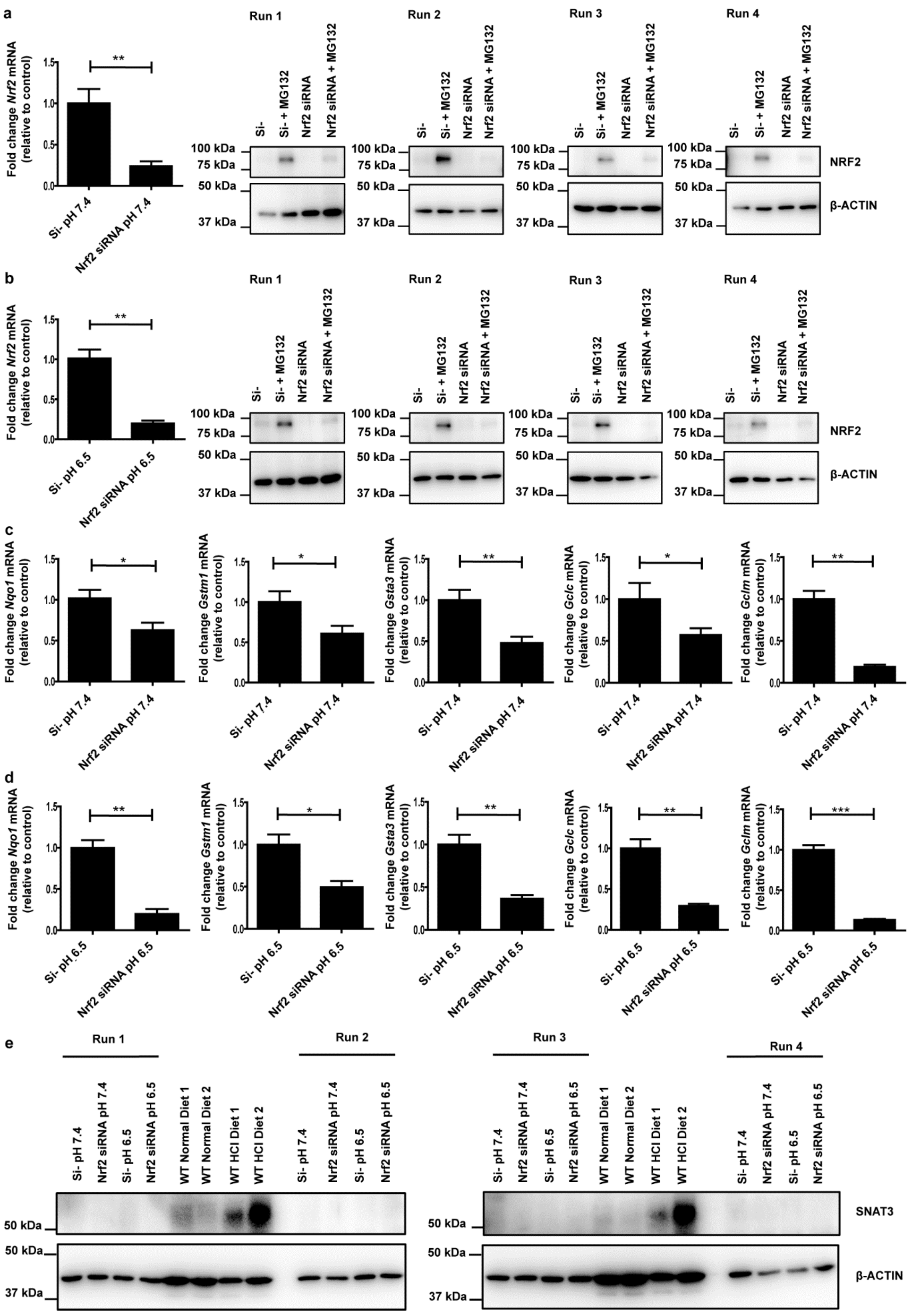

a
Run 1
Run 2
Run 3
Run 4
Fold change Nrf2 mRNA (relative to control)
**
Si- pH 7.4
Nrf2 siRNA pH 7.4
Si-
Si- + MG132
Nrf2 siRNA
Nrf2 siRNA + MG132
100 kDa
75 kDa
50 kDa
37 kDa
NRF2
β-ACTIN
b
Fold change Nrf2 mRNA (relative to control)
**
Si- pH 6.5
Nrf2 siRNA pH 6.5
c
Fold change Nqo1 mRNA (relative to control)
Fold change Gstm1 mRNA (relative to control)
Fold change Gsta3 mRNA (relative to control)
Fold change Gclc mRNA (relative to control)
Fold change Gclm mRNA (relative to control)
*
**
d
***
e
Si- pH 7.4
Nrf2 siRNA pH 7.4
Si- pH 6.5
Nrf2 siRNA pH 6.5
WT Normal Diet 1
WT Normal Diet 2
WT HCl Diet 1
WT HCl Diet 2
SNAT3
50 kDa
37 kDa
β-ACTIN

Supplemental Figure S2 Characterization of primary proximal convoluted tubular cells (PCT) as a model to investigate the biological mechanisms of action of the SNAT3 transporter. Confluent isolated PCT were either transfected with 20 nM siRNA targeting *Nrf2* or the non-targeting scrambled control, si-. Following incubation for 24 h, cells were exposed to media adjusted to pH 7.4 or 6.5 for a further 24 h. Due to its short half-life, in order to visualize the NRF2 protein, cells were exposed to the proteasome inhibitor MG132 at 10 µM for 2 h prior to lysis. QPCR and immunoblotting confirmed the depletion of *Nrf2* mRNA and protein following *Nrf2* siRNA in PCT cells cultured in medium adjusted to **a,** pH 7.4 and **b,** 6.5. QPCR confirmation of the depletion of the NRF2 target genes *Nqo1*, *Gstm1*, *Gsta3, Gclc* and *Gclm* following *Nrf2* siRNA in primary PCT cultured in medium adjusted to **c,** pH 7.4 and **d,** 6.5. **e,** SNAT3 protein levels following *Nrf2* siRNA in primary PCT cultured in medium adjusted to pH 7.4 and 6.5. Whole kidney lysates from WT normal diet and WT HCl diet animals were used as a positive control. 25 µg of sample and control protein were loaded for all immunoblots. The mRNA levels were normalized to *Ppia*. β-ACTIN was used as a house-keeping protein control. Data represent mean ± S.D. of n=4 independent PCT preparations. Statistical analysis for qPCR was performed with a student's paired t-test *$P \leq 0.05$; **$P \leq 0.01$.

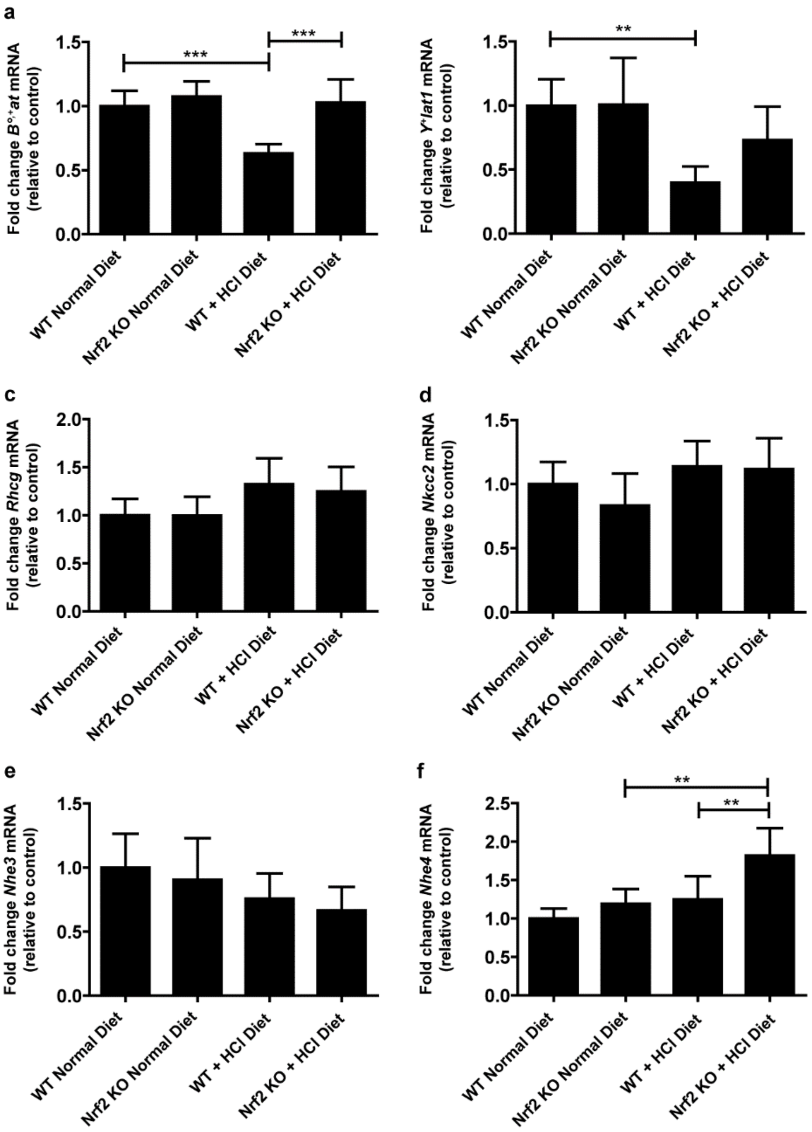

Supplementary Figure S3 The proton handling transporter *Nhe4* may help to compensate for the loss of *Snat3* in *Nrf2* KO kidney upon metabolic acidosis**.** WT and *Nrf2* KO mice were fed a normal diet or a HCl containing diet for 7 days. qPCR analysis of the kidney transporters for; amino acids, **a**-**b**, $B^{\circ,+}at$ and $y^{+}Lat1$, **c** -**d** for ammonium *Rhgc* and *Nkcc2*, and **e**-**f** the sodium/proton exchangers *Nhe3* and *Nhe4*. mRNA values were normalized to *Ppia*. Data represent mean ± S.D. of n=6 animals per group. Statistical analysis was performed with a one-way analysis of variance (with Tukey's post test); *P≤0.05; **P≤0.01; ***P≤0.001.

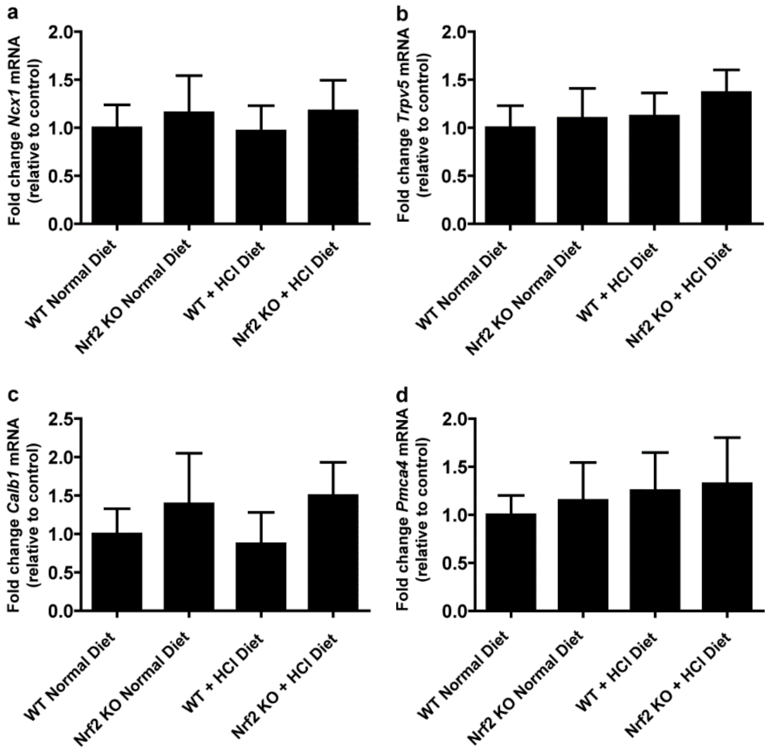

Supplementary Figure S4 Metabolic acidosis caused no changes in the mRNA expression of proteins involved in renal calcium handling in *Nrf2* KO kidney. WT and *Nrf2* KO mice were fed a normal diet or a HCl containing diet for 7 days. qPCR analysis for **a**, *Ncx1*, **b**, *Trpv5*, **c**, *Calb1* and **d**, *Pmca4a*. mRNA values were normalized to *Ppia*. Data represent mean ± S.D. of n=6 animals per group.

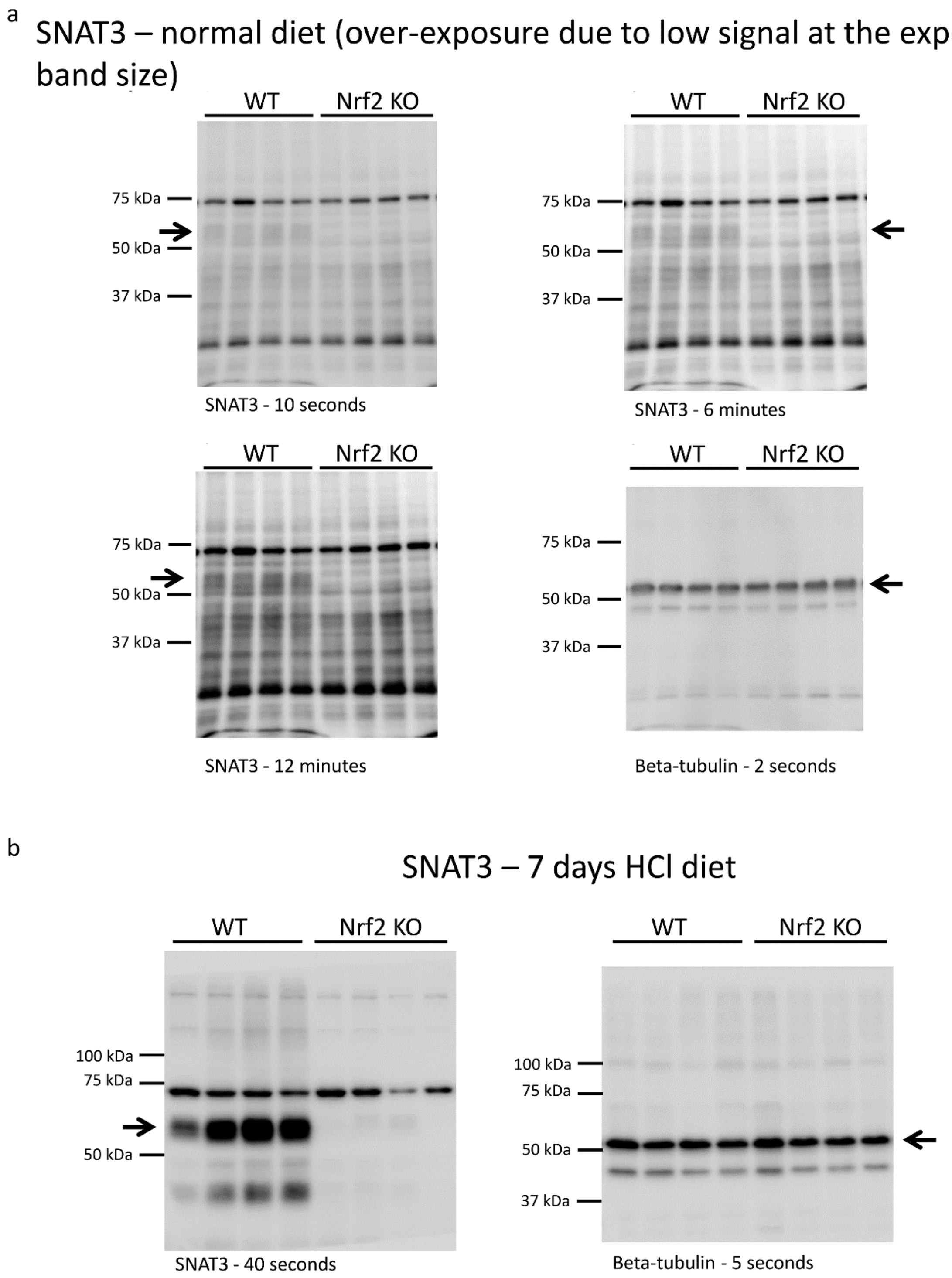

Supplementary Figure S5 Original full-length images used to create the cropped blots shown in Figure 1. **a**, SNAT3 expression under normal diet, **b**, SNAT3 expression upon 7 days HCl diet. Arrows indicate the band quantified by densitometry. Exposure time is shown below each western blotting image.

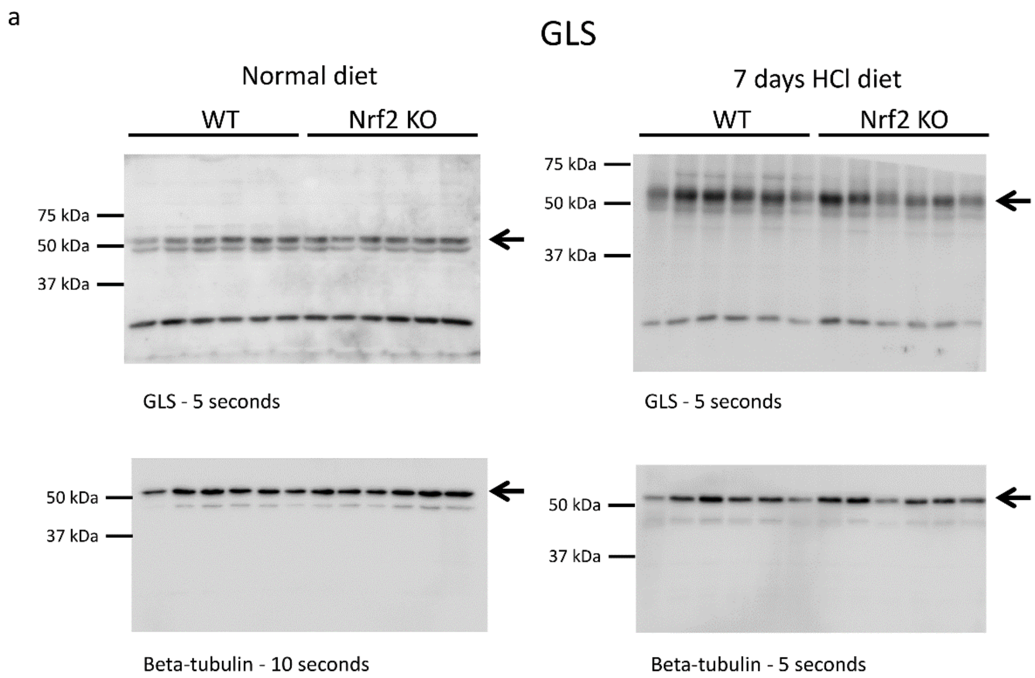

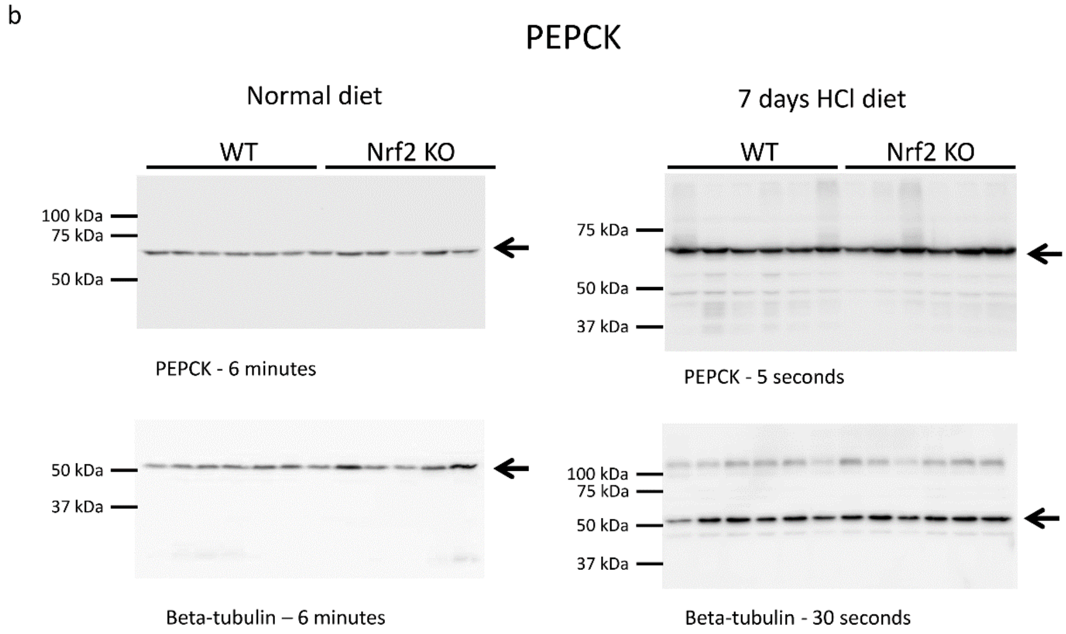

Supplementary Figure S6 Original full-length images used to create the cropped blots shown in Figure 2. **a**, GLS expression, **b**, PEPCK expression. Arrows indicate the band quantified by densitometry. Exposure time is shown below each western blotting image.

a 4F2HC – normal diet (over-exposure due to low signal at the expected band size)

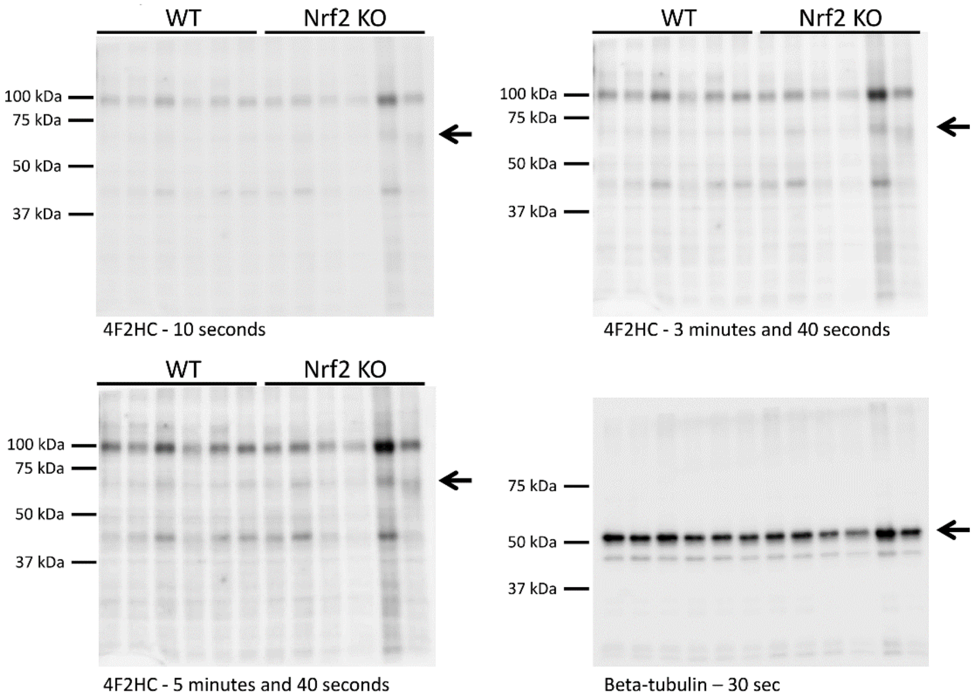

b

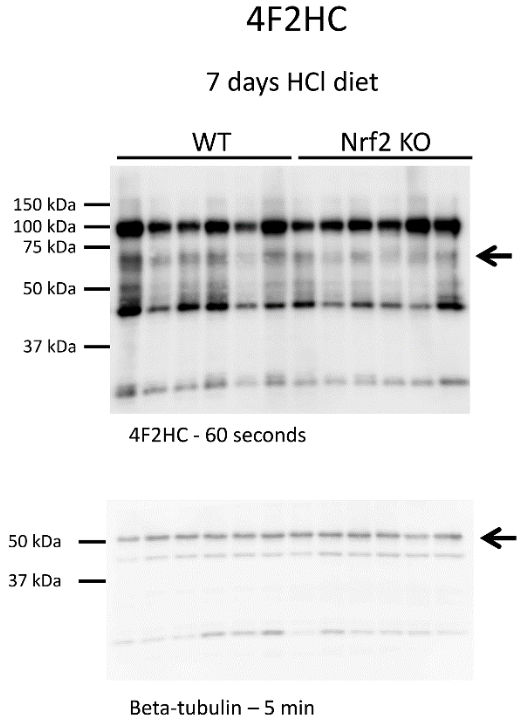

Supplementary Figure S7 Original full-length images used to create the cropped blots shown in Figure 4a-b. **a**, 4F2HC expression under normal diet, **b**, and upon 7 days HCl diet. Arrows indicate the band quantified by densitometry. Exposure time is shown below each western blotting image.

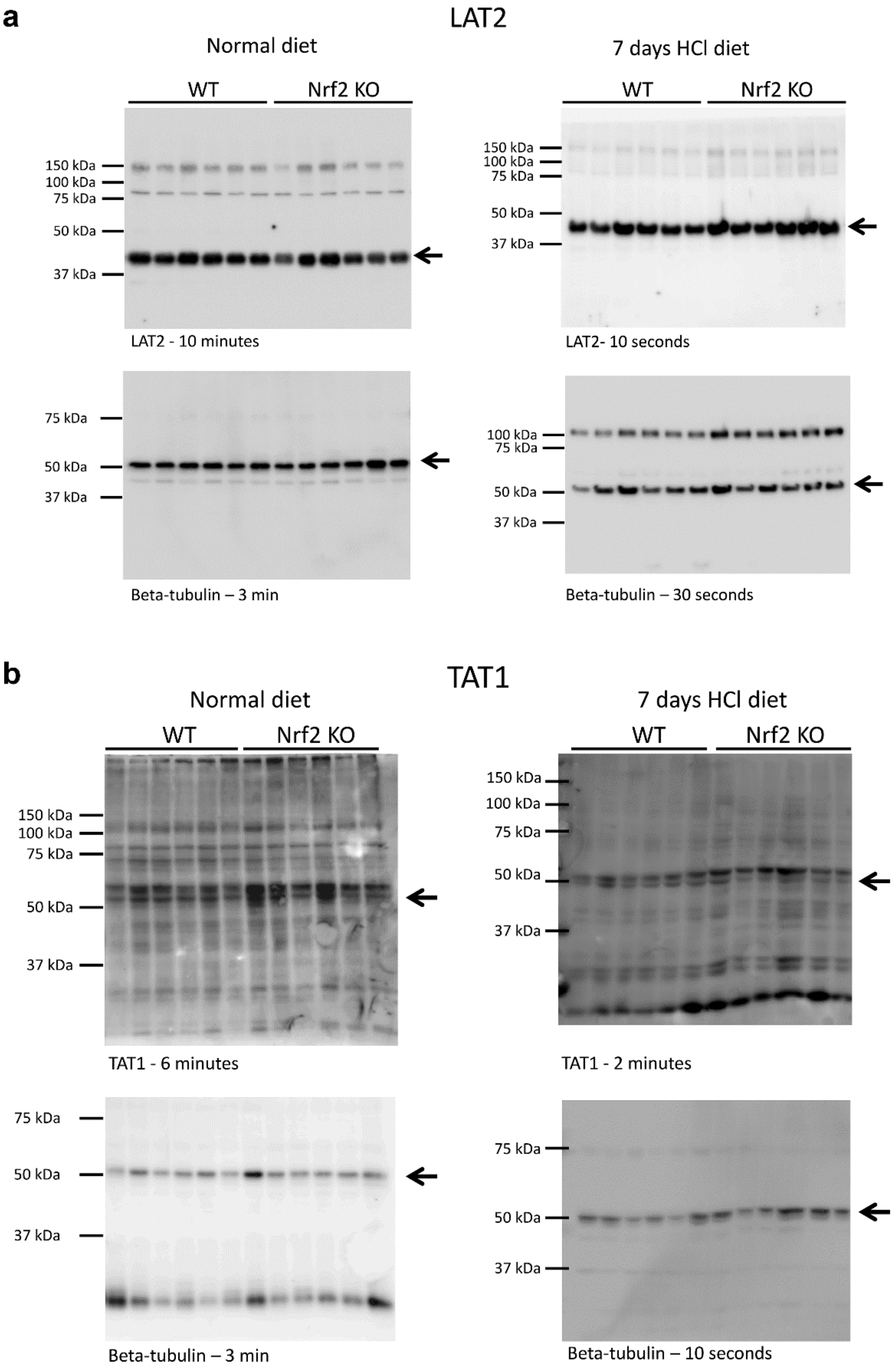

Supplementary Figure S8 Original full-length images used to create the cropped blots shown in Figure 4c-f. **a**, LAT2, **b**, TAT1 expression. Arrows indicate the band quantified by densitometry. Exposure time is shown below each western blotting image.

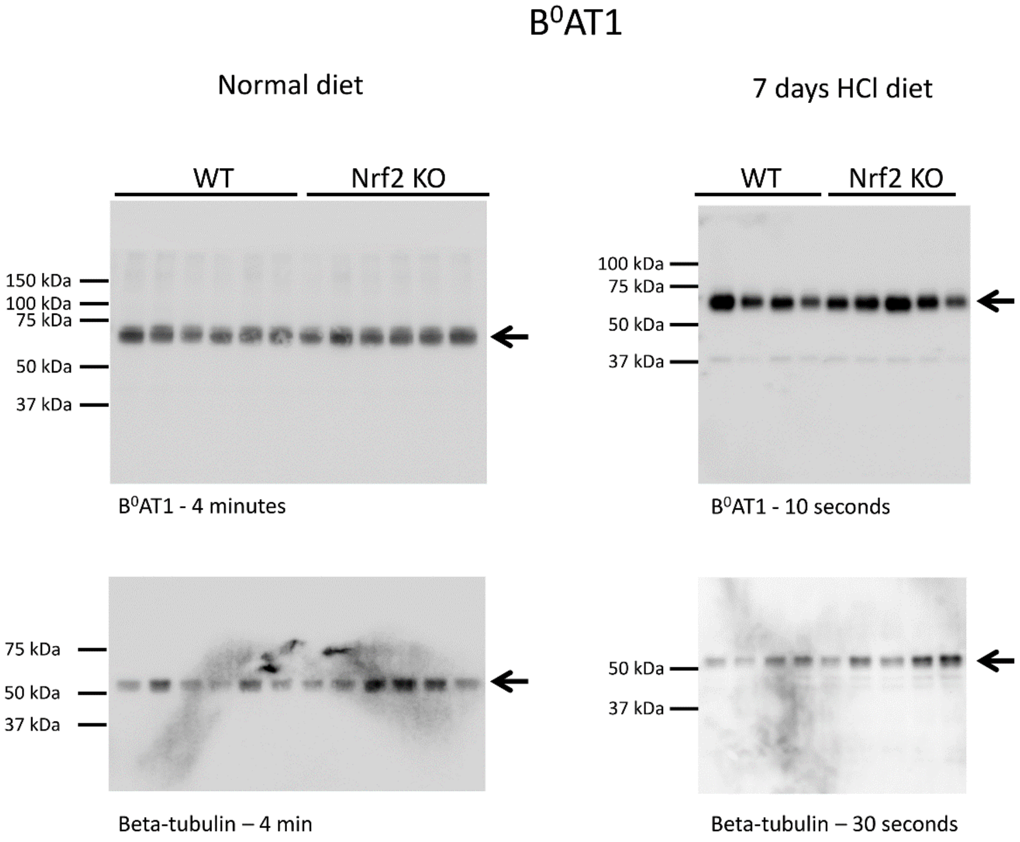

Supplementary Figure S9 Original full-length images used to create the cropped blots shown in Figure 4g-h. B^0AT1 expression. Arrows indicate the band quantified by densitometry. Exposure time is shown below each western blotting image.

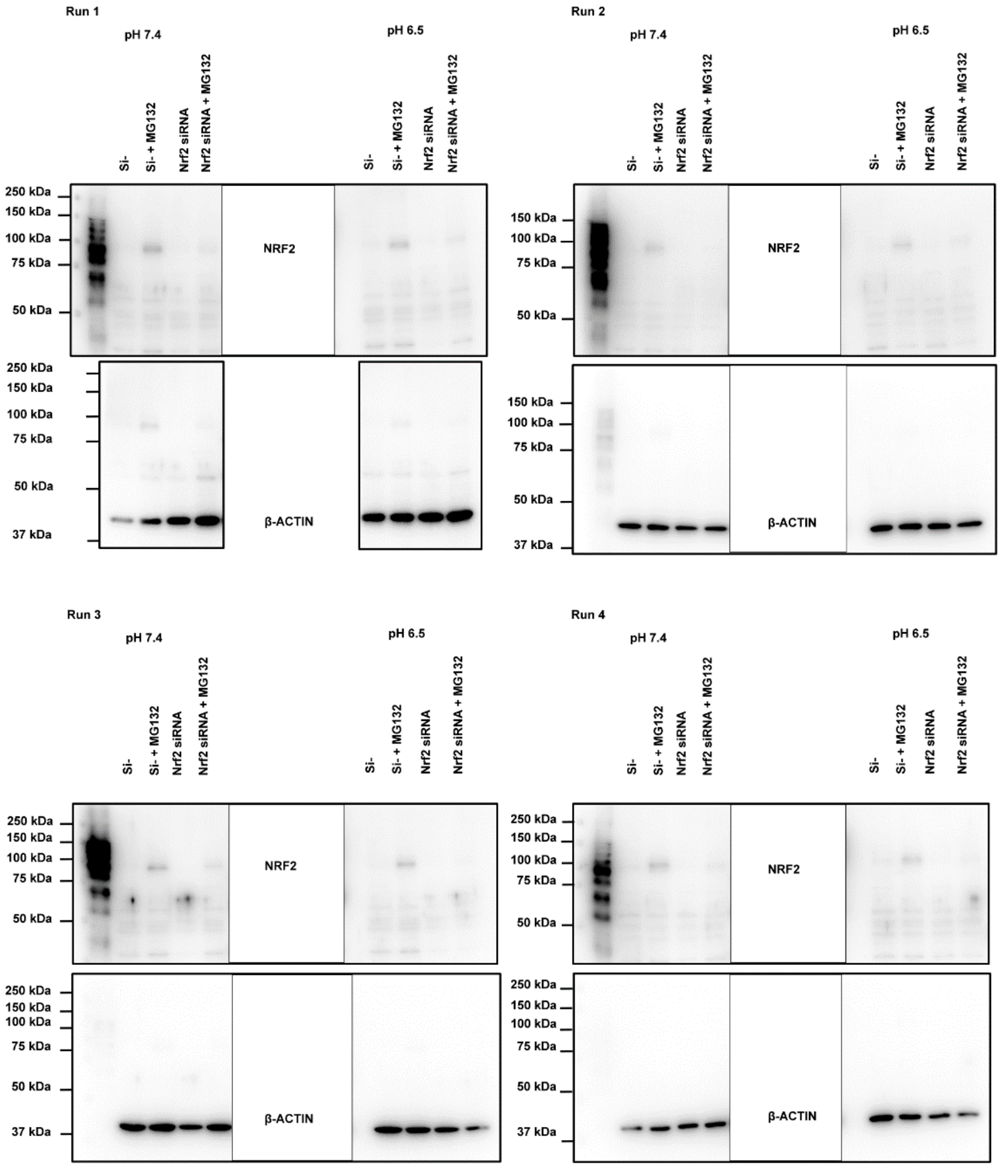

Supplemental Figure S10 Full length immunoblots from images presented in supplemental Figure S2a and b. Images confirm the depletion of NRF2 protein following *Nrf2* siRNA in PCT cells cultured in medium adjusted to pH 7.4 and pH 6.5. An amount of 25 µg of sample and control protein were loaded for all immunoblots. β-ACTIN was used as a house-keeping protein control. Images were acquired with the Imagequant LAS-4000 (Fujifilm) following exposure for 1 min (NRF2) and 10 sec (β-ACTIN).

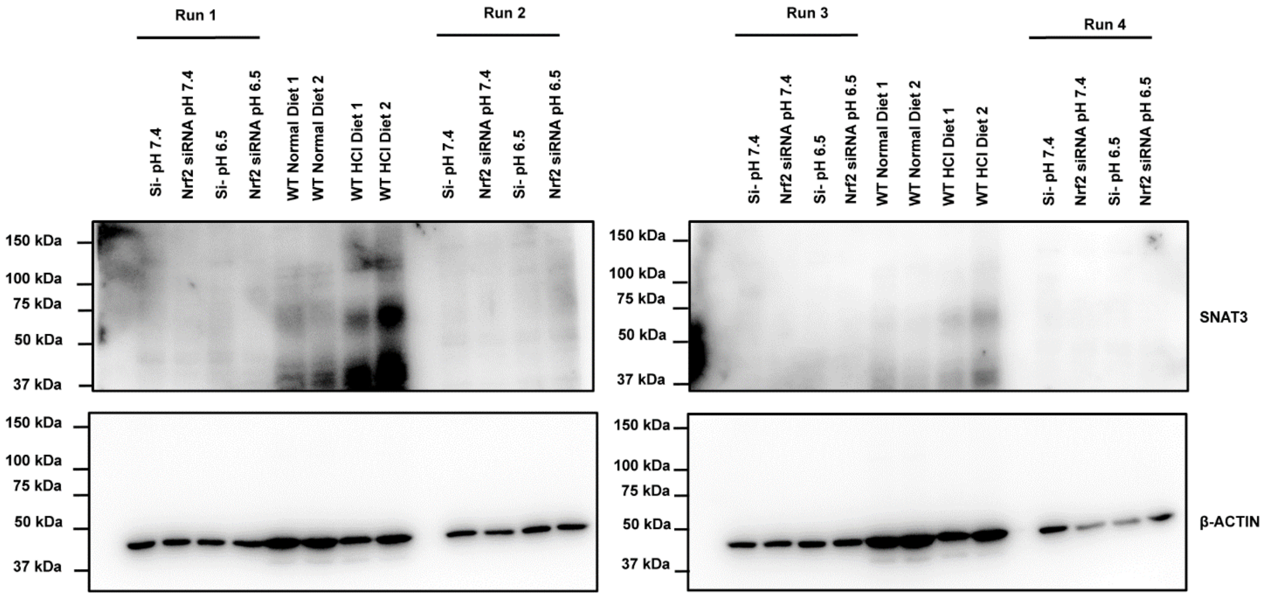

Supplemental Figure S11 Full length immunoblots from images presented in supplemental Figure S2e. Images show SNAT3 protein levels following *Nrf2* siRNA in primary PCT cultured in medium adjusted to pH 7.4 and pH 6.5. Whole kidney lysates from WT normal diet and WT HCl diet animals were used as a positive control. An amount of 25 µg of sample and control protein were loaded for all immunoblots. β-ACTIN was used as a house-keeping protein control. Images were acquired with the Imagequant LAS-4000 (Fujifilm) following exposure for 10 min (SNAT3) and 10 sec (β-ACTIN).

Supplementary Table S1. Urinary values in *C57Bl6* and *Nrf2 KO* mice under normal diet and a 2 or 7 days HCl load normalized to 24 h urine volume. Summary of urinary data obtained from WT and *Nrf2* KO mice fed a normal diet (control) or a HCl-containing standard diet for 2 and 7 days (acid). Data represent mean ± S.D. of n=6 animals per group. Statistical analysis was performed with a one-way analysis of variance (with Tukey's post test); *P≤0.05 for differences between genotypes for the same treatment and #P≤0.05 for differences between untreated and treated mice for the same genotype.

	Baseline		2 days HCl		7 days HCl	
	WT<br>*N = 12*	*Nrf2 KO*<br>*N = 12*	*WT*<br>*N = 6*	*Nrf2 KO*<br>*N = 6*	*WT*<br>*N = 6*	*Nrf2 KO*<br>*N = 6*
Urine						
Volume (ml/24 h)	2.4 ± 0.5	1.8± 0.5*	2.3 ± 0.7	2.5 ± 0.3#	2.1 ± 0.8	2.8 ± 0.9#
Creatinine excretion (µmol/24 h)	6.4 ± 0.7	5.7 ± 1.2	6.3 ± 1.2	6.8 ± 0.9	4.6 ± 1.9	6.5 ± 1.0
Urinary pH	6.3 ± 0.2	5.9 ± 0.3*	5.6 ± 0.1	5.4 ± 0.1	5.6 ± 0.5	5.3 ± 0.1
NH_4^+ (µmol/24h)	16.1 ± 13.1(6)	74.0 ± 52.7*(6)	98.2 ± 33.1#	80.5 ± 15.2#	244.2 ± 135.3#	131.6 ± 68.7#
TA (µmol/24h)	20.9 ± 10.8 (6)	61.4 ± 41.5*(6)	ND	ND	16.7 ± 15.2	15.4 ± 10.7#
NAE (µmol/24h)	37.0 ± 20.4 (6)	135.4 ± 91.6 *(6)	ND	ND	260.9 ± 148.3	146.9 ± 79.2
Pi (µmol/24h)	9.3 ± 3.2	18.3 ± 10.0*	30.9 ± 18.1#	28.6 ± 11.5#	4.1 ± 2.8#	3.7 ± 2.5#
Ca^{2+} (µmol/24h)	0.34 ± 0.18	0.44 ± 0.17	ND	ND	4.6 ± 1.3#	3.8 ± 1.3#
Na^+(µmol/24h)	32.0 ± 8.1	51.3 ± 13.6	ND	ND	50.8 ± 6.9	39.5 ± 6.1
Cl^- (µmol/24h)	63.0 ± 17.2	91.6 ± 30.9	ND	ND	259.4 ± 45.1#	237.2 ± 38.7#
K^+ (µmol/24h)	94.1 ± 27.4	124.1 ± 20.9	ND	ND	95.4 ± 12.6	87.1 ± 13.5

TA, titratable acidity; NAE, net acid excretion. Urinary NH_4^+, TA and NAE have been determined in 6 mice of each period.

Supplementary Table 2. Amino acids in plasma in wild-type and *Nrf2* KO mice after a 7 days HCl load.

**Amino acid (μM)**	**WT**	***Nrf2* KO**	**P-value**
Alanine	424.17 ± 185.51	484.5 ± 97.4	n.s.
Arginine	48 ± 11.64	59.05 ± 8.27	n.s.
Asparagine	40.68 ± 13.26	49.32 ± 15.44	n.s.
Aspartate	7.9 ± 3.51	9.38 ± 1.1	n.s.
Glutamine	509 ± 36.17	653.67 ± 79.55	0.002
Glutamate	34.57 ± 4.28	44.05 ± 4.7	0.009
Glycine	353.33 ± 115.87	669.17 ± 698.89	n.s.
Histidine	69.83 ± 8.06	88.72 ± 12.04	0.026
Isoleucine	86.67 ± 21.42	92.18 ± 31.57	n.s.
Leucine	139.33 ± 16.72	143.33 ± 24.73	n.s.
Lysine	206.67 ± 43.07	256 ± 39.58	n.s.
Methionine	58.82 ± 20.41	64.9 ± 10.38	n.s.
Phenylalanine	73.13 ± 11.18	95.43 ± 17.74	0.026
Proline	104.78 ± 49.3	123.77 ± 33.62	n.s.
Serine	166.5 ± 63.7	169.33 ± 34.03	n.s.
Threonine	258.17 ± 79.58	226.33 ± 68.95	n.s.
Tryptophan	66.22 ± 14.14	83.1 ± 23.72	n.s.
Tyrosine	65.77 ± 17.93	78.17 ± 24.4	n.s.
Valine	215 ± 34.89	234.67 ± 40.12	n.s.
α-aminoadipic acid	15.25 ± 4.86	13.67 ± 2.81	n.s.
γ-aminobutyric acid	8.17 ± 1.3	9.85 ± 2.3	n.s.
Anserine	1.07 ± 1.2	0.88 ± 0.79	n.s.
β-Alanine	1.24 ± 0.54	1.26 ± 0.28	n.s.
Citrulline	52.15 ± 11.81	59.55 ± 11.17	n.s.
Cystine	9.18 ± 4.63	4.46 ± 2.69	n.s.
GABA	0.2 ± 0.07	0.44 ± 0.17	0.015
1-M-histidine	4.2 ± 0.86	4.66 ± 0.78	n.s.
3-M-histidine	3.64 ± 0.88	8.52 ± 0.98	0.002
OH-Proline	31.63 ± 12.17	27.05 ± 8.98	n.s.
Ornithine	36.2 ± 13.78	54.23 ± 16.37	n.s.

Values as mean ± SD, P-value calculated with the Mann-Whitney test
n.s.: not significant differences

Supplementary Table 3. Amino acids in kidney in wild-type and *Nrf2* KO mice after a 7 days HCl load.

**Amino acid (µmol/kg kidney)**	**WT**	***Nrf2* KO**	**P-value**
Alanine	1187 ± 233.96	1160.5 ± 243.83	n.s.
Arginine	44.6 ± 5.68	38.65 ± 6.35	n.s.
Asparagine	97.9 ± 12.76	116.5 ± 22.66	n.s.
Aspartate	1902 ± 481.24	1831 ± 364.58	n.s.
Glutamine	678 ± 149.25	727.5 ± 210.15	n.s.
Glutamate	3780 ± 431.83	3870 ± 376.62	n.s.
Glycine	6845 ± 675.21	5205 ± 590.86	0.004
Histidine	184.75 ± 35.12	116.2 ± 18.48	0.004
Isoleucine	93.9 ± 21.11	98.35 ± 14.39	n.s.
Leucine	221.95 ± 19.82	225.65 ± 20.94	n.s.
Lysine	65.9 ± 11.84	63.05 ± 9.14	n.s.
Methionine	61.66 ± 37.59	66.45 ± 15.11	n.s.
Phenylalanine	75.2 ± 8.95	108.7 ± 22.05	0.004
Proline	102.85 ± 20.03	131.1 ± 30.93	n.s.
Serine	564 ± 118.08	566 ± 67.93	n.s.
Threonine	367 ± 85.04	293.75 ± 55.6	n.s.
Tryptophan	13.13 ± 6.08	20.77 ± 8.29	n.s.
Tyrosine	93.05 ± 10.72	107.35 ± 37.47	n.s.
Valine	220.85 ± 21.89	263.9 ± 25.23	0.026
α-aminoadipic acid	126 ± 16.22	98.55 ± 25.39	n.s.
γ-aminobutyric acid	16.52 ± 4.68	15.65 ± 3.11	n.s.
Anserine	12.52 ± 0.98	15.37 ± 1.7	0.041
β-aminoisobutyric acid	11.05 ± 7.02	23.84 ± 7.93	n.s.
β-Alanine	2.7 ± 0.31	2.35 ± 0.22	0.009
Carnosine	2.91 ± 0.38	4.25 ± 1.21	0.015
Citrulline	19.95 ± 3.87	25.36 ± 4.87	n.s.
Cystine	325.6 ± 95.42	235.75 ± 55.86	n.s.
GABA	26.57 ± 8.91	44.85 ± 9.31	n.s.
1-M-histidine	15.66 ± 3.53	15.55 ± 1.87	n.s.
3-M-histidine	79.65 ± 25.13	48.32 ± 17.62	0.009
Homocystine	0.03 ± 0.03	0.02 ± 0.01	n.s.
OH-Proline	56.05 ± 17.24	41 ± 8.59	n.s.
Ornithine	15.55 ± 10.64	17.01 ± 9.52	n.s.

Values as mean ± SD, P-value calculated with the Mann-Whitney test
n.s.: not significant differences

Supplementary Table 4. Primers used for qPCR analysis of mouse kidney tissue.

Gene	Primer	Sequence
mouse *Snat1* (*Slc38a1*)	sense	TCCTTCAGCCATAAAATCCCTC
	anti-sense	CCACTCGTGTAGCCAAGATAC
mouse *Snat2* (*Slc38a2*)	sense	CCGTCTGCCTTCTACATCAAG
	anti-sense	CCCAATCCAGCACAATCAAG
mouse *Snat3* (*Slc38a3*)	sense	TCGGCTACCTGGGTTACTC
	anti-sense	GGGAACAGAACAATCGGAACTG
mouse *Snat4* (*Slc38a4*)	sense	CCTCGTGCCTACCATCAAATAC
	anti-sense	AGACCAAAGCCCCAATCTTC
mouse *Snat7* (*Slc38a7*)	sense	GTAGTGTACCCGTCTTCAACAG
	anti-sense	AAGGTTAGGAAGCCACAGATG
mouse *Pepck* (*Pck1*)	sense	CTGCATAACGGTCTGGACTTC
	anti-sense	CAGCAACTGCCCGTACTCC
mouse *Gls* (*glutaminase*)	sense	GTGGTTTCTGCCCAATTACTG
	anti-sense	CCCAGCAACTCCAGATTTTG
mouse *Tat1* (*Slc16a10*)	sense	CCCCATCGTGAGTGTCTTC
	anti-sense	TCCATAGGTGAGGTACAGAGG
mouse *4f2hc* (*Slc3a2*)	sense	ACCTCACTCCCAACTACCAG
	anti-sense	ATCAGCTTTCCCACATCCC
mouse *Lat1* (*Slc7a5*)	sense	TCACTACCCTCTCTACCAACC
	anti-sense	TGAACAGAGACCCATTGACAG
mouse *Lat2* (*Slc7a8*)	sense	AAAGAGATCGGATTGGTCAGC
	anti-sense	ATCCAGACAATGAGAGCAAGG
mouse y+lat1 (Slc7a7)	sense	CTTTATCTACGCTGGAAGGACC
	anti-sense	GATGAGGGAGTTGATGGTGTC
mouse *Bo,+at* (*Slc7a9*)	sense	GCTCTTGCAGTCCCAGGCT
	anti-sense	GGGACTACCCAAGATGCTGGA
mouse *B(o)at1* (*Slc6a19*)	sense	CTCATCCTTCTGGTGTTCGAG
	anti-sense	ATGAAGGACACGAACATGGAG
mouse *Nrf2(Nfe2l2)*	sense	CAGCATGTTACGTGATGAGG
	anti-sense	GCTCAGAAAAGGCTCCATCC
mouse *Nqo1*	sense	TTTAGGGTCGTCTTGGCAAC
	anti-sense	GTCTTCTCTGAATGGGCCAG
mouse *Gclc*	sense	GGGGTGACGAGGTGGAGTA
	anti-sense	GTTGGGGTTTGTCCTCTCCC
mouse *Gclm*	sense	AATCAGCCCCGATTTAGTCAG
	anti-sense	CGATCCTACAATGAACAGTTTTGC
mouse *Car3*	sense	TGGACGGAGTAAAATACGCTG
	anti-sense	AATCTGGAACTCGCCTTTCTC

mouse *Nhe3* (*Slc9a3*)	sense	CACACCCCGCCCATCTACT
	anti-sense	CCAGGCATACAGCACTGACATT
mouse *Nhe4* (*Slc9a4*)	sense	GAGGATACAGGGAATCAAGCG
	anti-sense	CTTGCTTCTCACTTGTTTGGG
mouse *Rhcg*	sense	AGTGACCTGGATCCTCTACC
	anti-sense	GAACTGGCTGAATTGAAACTGG
mouse *Nkcc2* (*Slc12a1*)	sense	TGCTAATGGAGATGGGATGC
	anti-sense	CAGGAGAGGCGAATGAAGAG
mouse *Ncx1* (*Slc8a1*)	sense	TCCATCCAGTAGACTTCGTGAT
	anti-sense	CCAAGCAATTCCTTACAGAGTGA
mouse *Trpv5*	sense	TGCTGCTATAATGCTGATGGAG
	anti-sense	GCACGGACTAGGTTCACATTCT
mouse *Calb1* (*Calbindin d28k*)	sense	AGAACTTGATCCAGGAGCTTC
	anti-sense	CTTCTGTGGGTAAGACGTGAG
mouse *Pmca4* (*Atp2b4*)	sense	GGATTGGAGAACTTTTGTGGG
	anti-sense	ATCTCGGCAAGGTCAATCTC
mouse *Cyp2a5*	sense	GGACAAAGAGTTCCTGTCACTGCTTC
	anti-sense	GTGTTCCACTTTCTTGGTTATGAAGTCC
mouse *Gsta3*	sense	GGTTCCTGGTTTGTTCCTTG
	anti-sense	CTATGGGAAGGACATGAAGGAG
mouse *Gstm1*	sense	ATACTGGGATACTGGAACGTCC
	anti-sense	AGTCAGGGTTGTAACAGAGCAT
mouse *Dpys*	sense	TGTGACTATAGCCTGCATGTG
	anti-sense	CGGGCAAGGGTTTTCATTTC
mouse *Kim1* (*Havcr1*)	sense	ACTAAGGGCTTCTATGTTGGC
	anti-sense	AGCTTCAATCTTAGAGACACGG
